# Structural Templation of MOF-Derived Zirconia Nanoparticles

**DOI:** 10.1021/acsami.5c14965

**Published:** 2025-11-14

**Authors:** Joshua A. Powell, Maxwell W. Terban, Jiaqi Zhang, Songsheng Tao, Jingwei Hou, Simon J. L. Billinge, Hong-Cai Zhou

**Affiliations:** † Department of Chemistry, Texas A&M University, College Station, Texas 77843, United States; ‡ School of Chemical Engineering, 1974 The University of Queensland, St Lucia 4072, Australia; § 28326 Max Planck Institute for Solid State Research, Heisenbergstr. 1, Stuttgart 70569, Germany; ∥ Department of Applied Physics and Applied Mathematics, 5798 Columbia University, New York, New York 10027, United States; ⊥ Department of Materials Science and Engineering, Texas A&M University, College Station, Texas 77843, United States

**Keywords:** metal−organic framework, templated synthesis, zirconia, X-ray total scattering, transmission electron microscopy

## Abstract

Templated synthesis is an important avenue for the development and synthesis of porous materials, as it provides a high level of control over the resulting structure. However, this control is difficult to achieve at the atomic level for poorly crystalline or noncrystalline materials, such as metal–organic framework (MOF)-derived carbons. We report the carbonization of three zirconium-based MOFs with different framework and inorganic building unit structures to form zirconia nanoparticles in a carbon matrix. Using a combination of X-ray diffraction, X-ray total scattering, and transmission electron microscopy, we found that the extended Zr-oxo chains of MIL-140C-bpy facilitate the formation of larger and more ordered zirconia nanoparticles. In contrast, the discrete Zr_6_-oxo clusters of UiO-67-bpy and Zr-ABTC result in smaller and differently structured nanoparticles.

## Graphical abstract

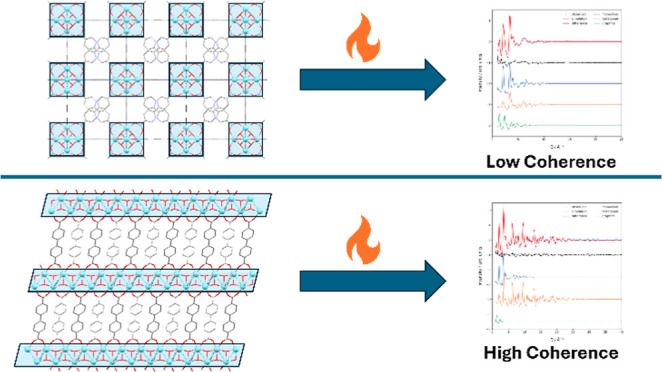

## Introduction

For decades, chemists and materials scientists have used templated synthesis to design new materials with unique chemical structures or morphologies.^1–3^ In the field of inorganic chemistry, this has mainly involved the use of sacrificial hard templates such as carbon nanotubes^4,5^ or surfactants^6^ to obtain a desired morphology. Likewise, in polymer chemistry, hard templates such as metal oxides or soft templates such as small molecules with strongly interacting functional groups are often used to obtain a desired morphology or impart porosity.^2,7,8^

More recently, MOF research has begun to similarly use the concept of structural templation to design functional materials.^9–12^ Templates such as microfluidic channels have been used to template the macroscopic morphology of large single crystals into unusual shapes that do not occur naturally, enabling the use of MOFs in functional devices by eliminating the consideration of crystal packing and grain boundaries.^11^ Likewise, addition of truncated linkers to MOF synthesis as modulators can similarly control the morphology of crystallites by inhibiting growth on a particular face.^13^ On the microscopic scale, sacrificial templation has become a common strategy to generate hierarchical porosity in MOFs, with templates such as nanoparticulate polystyrene or metal being used to template the formation of larger pores in the crystallites.^9,14,15^ Postsynthetic linker or metal exchange can also be treated as a templated synthesis, in which one linker or metal is used to template the formation of a desired structure, and is then replaced by another functional component postsynthetically.^16,17^ This templating approach can enable the formation of structures that are inaccessible via direct synthesis and substantially expands the diversity of MOFs that can be synthesized.

MOFs have also been used as templates themselves, as their highly ordered, porous crystalline structure presents an attractive platform for the design of functional materials. Simple MOFs have been used as structural templates for other, more complex MOFs through strategies such as linker substitution,^18,19^ and as morphological templates for other porous materials such as porous polymers or metal nanoparticles.^10,20,21^ Unfortunately, it is difficult to template desirable structural features in a highly controlled manner into poorly crystalline or noncrystalline materials due to their lack of long-range order, especially when it comes to ordering at the atomic level. This lack of structural coherence and poorly defined atomic ordering poses a significant challenge in predictably imparting desirable structural features into the templated material.^22^

A recent advance in the field of MOF and solid-state chemistry is the development of MOF-derived carbons (MOFdCs).^23–26^ Upon heating under nonoxidizing conditions, the organic linkers of the MOF are transformed into a porous carbon matrix embedded with inorganic nanoparticles derived from the inorganic building units (IBUs) of the MOF.^23^ As with any crystalline-to-amorphous transition, much of the structural information of the MOF is lost during the calcination, as the well-defined structure of the MOF is largely destroyed. While this removes a key advantage of MOF materials, their structural order and ease of structural control, it greatly enhances the stability of the resulting material, which can provide great benefits for using the materials as catalysts or energy storage materials under harsh physical or chemical conditions.^27–31^ Additionally, MOFdCs can be used to form a range of inorganic nanoparticles that are ultrasmall or otherwise unstable or difficult to synthesize under standard conditions.^32–37^

Calcination conditions can play a significant role in determining the structure of the final material. For example, calcination of a MOF in an air environment encourages combustion of the organic components, leading to a porous metal oxide product.^38,39^ In contrast, calcination under nitrogen or other nonoxidizing gases preserves the organic components as a graphitic porous carbon matrix in which metal oxide nanoparticles are embedded.^35,40^ Similarly, calcination of MOFs that do not contain oxygen in the linker or IBU can result in metal nitride or metal nanoparticles in the carbon matrix if the gas environment is rigorously air-free, while oxides are formed if even trace air is allowed into the calcination.^32^

Additionally, template structure plays a role in the type of products that are formed. The ZIF-8/ZIF-67 family of zeolitic imidazolate frameworks is a common template for forming MOFdCs, as the nitrogen rich imidazolate linkers are transformed into nitrogen-doped carbon upon calcination.^41–44^ This is especially useful, as the N-doped carbon can bind single metal atoms or ultrasmall metal clusters to the carbon matrix, which can be applied as catalysts for various industrially relevant processes.^43–46^

The Zhou group has previously noted that calcination of UiO-66 produces zirconia nanoparticles that are ultrasmall and exhibit an average atomic structure that best resembles the cubic phase of zirconia.^47^ However, X-ray and neutron total scattering experiments indicated that the local structure of the nanoparticles could be best described by the tetragonal phase of zirconia.^47^ Intrigued by this, we set out to further investigate the structures of MOF-derived zirconia nanoparticles by calcining three MOFs with different structures, with the ultimate goal of understanding the templating effect that the MOF and IBU structures have on the structure of the resulting zirconia nanoparticles. Herein we show that IBUs with extended chains produce larger and more ordered nanoparticles, while IBUs that are comprised of discrete clusters produce smaller and less ordered nanoparticles. While differentiating between individual phases may not be appropriate at such small length scales, we show that the local structure will appear less symmetrical in zirconia nanoparticles derived from discrete clusters due to the high concentration of defects, even if the structure appears to be of higher symmetry at longer length scales.

## Results and Discussion

To study the effect of MOF and IBU template structure on the structure of the zirconia nanoparticles, we selected three template Zr-MOF structures to calcine, namely UiO-67-bpy, Zr-ABTC, and MIL-140C-bpy (Table 1). UiO-67-bpy and MIL-140C-bpy are related structures, both formed with the same ditopic 2,2′-bipyridine-5,5′-dicarboxylic acid linker and thus exhibiting similar total carbon contents. The key differences between these two MOFs lie in the structures of the IBUs and the overall symmetries of the MOFs (Figure 1a,b). Where UiO-67-bpy is a cubic MOF containing Zr_6_-oxo IBUs with *O*_*h*_ symmetry, MIL-140C-bpy is a monoclinic MOF with *C*_2_ symmetric Zr-oxo chains. Zr-ABTC was chosen as an intermediate structure between the other two MOFs. While Zr-ABTC contains the same Zr_6_-oxo clusters as UiO-67-bpy, the geometric constraints imposed by the tetratopic 3,3′,5,5′-azobenzene-tetracarboxylic acid linker enforce a monoclinic symmetry in the MOF overall (Figure 1c). The different linker geometry and cluster connectivity also results in a lower decomposition temperature and total carbon content in Zr-ABTC, although the carbon/nitrogen ratio remains similar (6:1 vs 8:1). The latter property is particularly important due to the ability of nitrogen sites in N-doped carbon to coordinate single metal atoms or ultrasmall metal clusters.^48,49^ By comparing these three MOFs, the effects of both IBU structuring and overall MOF structuring on zirconia formation can be examined. The template MOFs were successfully synthesized using procedures adapted from the literature as determined by powder X-ray diffraction (PXRD) (Figure S1).^50–52^ No crystalline phase impurities could be observed in the pristine MOFs. The crystallites of the MOFs ranged from 0.2 to 20 μm in size, depending on the MOF (Figure S2).

**Table 1:** Formulas, Carbon Content, Decomposition Temperatures, and Crystallite Sizes of Template MOFs

MOF	formula unit	carbon content^a^ (wt %)	decomposition temperature (°C)	crystallite size (μm)
UiO-67-bpy	Zr_6_O_32_C_72_N_12_H_40_	40.55	480	0.2–0.5
Zr-ABTC	Zr_6_O_32_C_32_N_4_H_12_	25.42	425	20–50
MIL-140C-bpy	ZrO_5_C_12_N_2_H_6_	41.25	525	2–5

a Carbon content is estimated based on the formula unit of a “perfect crystal” with no defects and no adsorbed solvent. In reality, the true carbon content at the decomposition temperature may be higher or lower based on the presence of missing linker or missing cluster defects, the presence of residual adsorbed solvent, and slow linker thermolysis at subdecomposition temperatures.

**Figure 1:**
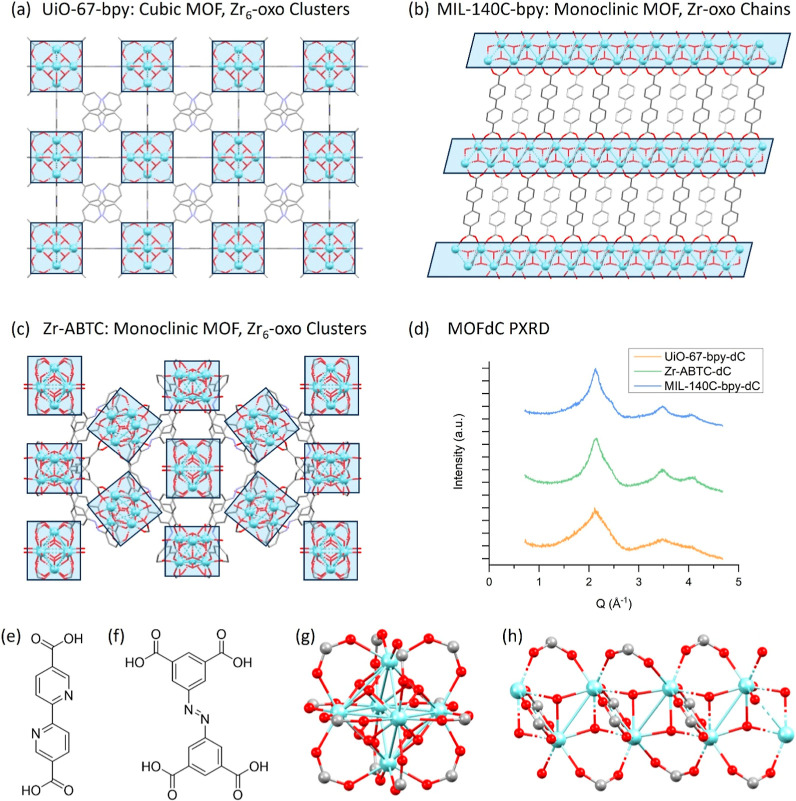
Crystal structures of (a) UiO-67-bpy, (b) MIL-140C-bpy, and (c) Zr-ABTC with the cluster structure highlighted; (d) powder X-ray diffraction patterns of MOF-derived carbons following calcination at 550 °C; (e) structure of H_2_bpydc linker used in UiO-67-bpy and MIL-140C-bpy; (f) structure of H_4_ABTC linker used in Zr-ABTC; (g) structure of discrete Zr_6_-oxo clusters occurring in UiO-67-bpy and Zr-ABTC; (h) structure of Zr-oxo chain occurring in MIL-140C-bpy.

The template MOFs were calcined in a nitrogen environment at 550 °C, at least 25 °C above the decomposition temperatures of the MOFs (Figure S3). Upon calcination, the white or orange MOF powders were transformed into black or dark brown powders, indicating successful carbonization of the organic linkers (Figure S4). There were no visible white spots, even under magnification with an optical microscope, indicating that the zirconia particles were very small. Lab-scale PXRD suggested that no crystalline domains of the template framework remained after calcination (Figure 1d). Elemental microanalysis (CHNS) indicated that the C/N ratios of UiO-67-bpy-dC and MIL-140C-bpy-dC were similar, while Zr-ABTC-dC had a lower nitrogen content, likely due to evolution of nitrogen gas from the azo group upon decomposition (Table S1).

All three samples showed broad peaks in the *Q* range of 1–6 Å^–1^ in agreement with literature zirconia powder patterns. Although Rietveld refinements were attempted to quantify the phase mass fractions, the broadness of the peaks, indicative of ultrasmall crystalline domains and limited long-range order, largely precludes such quantitative analysis.^53,54^ The ultrasmall domains and lack of long-range order are in agreement with previous studies on the calcination of similar MOFs.^47^ Therefore, we must limit our analysis of the average structure to qualitative observations.

There were several differences observed in the average structures of the three different MOFdCs. First, the zirconia NPs in UiO-67-bpy-dC and Zr-ABTC-dC best resemble high symmetry (cubic or tetragonal) zirconia. While it is difficult to distinguish the cubic and tetragonal phases with PXRD, especially when the peaks are so broad, there is no evidence of the splitting of the peak at *Q* = 2.2 Å^–1^ that is characteristic of monoclinic zirconia. On the other hand, the aforementioned peak contains a shoulder in the pattern of MIL-140C-bpy-dC which may be indexed by monoclinic zirconia, likely in combination with some higher symmetry structure. In addition to phase identification, there are differences in the broadness of the peaks in the PXRD patterns. The peaks become sharper in the order UiO-67-bpy-dC < Zr-ABTC-dC < MIL-140C-bpy-dC. It is unclear from the patterns whether this is indicative solely of differences in crystallite sizes or whether the degree of atomic ordering also plays a role.

The significant uncertainty caused by the broadness of the PXRD peaks necessitates the use of complementary methods to obtain more meaningful and quantitative information. Raman spectroscopy, a simple and more accessible technique for zirconia phase identification, is unsuitable for analysis of MOFdCs due to the strongly absorbing carbon, the low zirconia concentration, and the limited penetration depth of the technique (Figure S7).^55–57^ Thus, we turned to X-ray total scattering pair distribution function (PDF) analysis to further investigate the identity of the zirconia species. A key limitation in using Rietveld analysis of PXRD data is that it loses sensitivity when the structure contains long-range ordering, so the reliability of Rietveld refined models is significantly decreased or completely insensitive to the structure of very small particles. The increased surface-to-volume and defect density further decrease the validity of models that assume long-range ordering. On the other hand, PDF is sensitive to the local interatomic environments independent of the presence or absence of crystallinity or long-range order.^58^ Additionally, cubic and tetragonal zirconia are difficult to distinguish in PXRD, especially when the peaks are broad and with low intensity, due to the closeness of the major peaks in the two species. PDF highlights differences in the interatomic distances within the structure of the material. Although cubic and tetragonal zirconia also have similar structures, our simulations of literature structures (ICSD coll. codes 66781 and 89429) suggest that the PDF of tetragonal zirconia exhibits features not present in the PDF of cubic zirconia (Figures S8 and S9), which may allow for differentiation of the two phases (Figure 2c).

**Figure 2:**
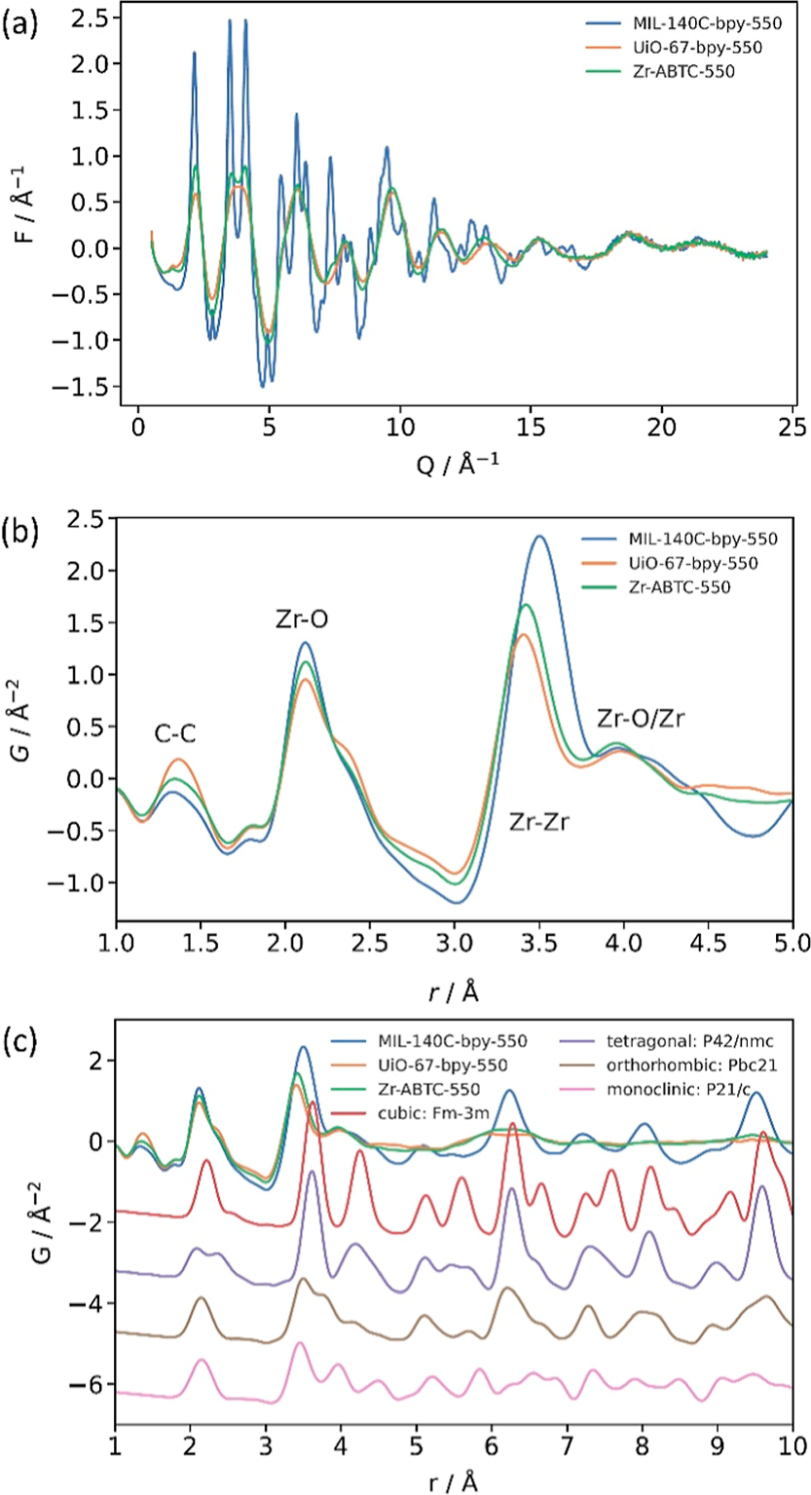
(a) *F*(*Q*) functions for each MOFdC and (b) *G*(*r*) functions for each MOFdC between 1 and 5 Å and (c) between 1 and 10 Å, compared with known phases of zirconia.

We first conducted a holistic analysis of the *F*(*Q*) and *G*(*r*) functions for each of the three MOFdCs. There are notable differences between the materials, both with respect to domain size and the degree of atomic structuring. In the *F*(*Q*) functions for each sample (Figure 2a), UiO-67-bpy-dC and Zr-ABTC-dC both exhibit significantly increased damping at high *r* compared to MIL-140C-dC. This indicates that some of the differences in peak broadness observed in the PXRD are simply a consequence of zirconia domain size. The zirconia domain sizes appear to be on the order of 15 Å for UiO-67-bpy-dC and Zr-ABTC-dC, less than half the domain sizes observed for MIL-140C-bpy-dC. However, as seen by the relative sharpness and definition of the peaks in the *F*(*Q*) function for each sample, the degree of atomic structuring is also notably different between the three materials. This indicates that the differences in peak broadness observed in the PXRD are not purely a consequence of domain size. UiO-67-bpy-dC is the least ordered of the three MOFdCs, while MIL-140C-bpy-dC is the most ordered, which is in agreement with the qualitative observations regarding peak width in the PXRD patterns. Importantly, there does not appear to be a substantial amount of unmodified IBU structuring present in the MOFdCs (Figures S11–S13), however the structure of the IBUs appears to have a templating effect on the degree of long-range order present. The extended Zr-oxo chains of MIL-140C-bpy may play a significant role in the templation of longer-range ordered nanoparticles, compared to the discrete Zr_6_-oxo clusters of the other two MOFs that tend to produce smaller and less-well-ordered nanoparticles.

Our qualitative analysis of the *G*(*r*) function allowed us to assign interatomic distances to several low *r* peaks and note several differences between the three MOFdCs (Figure 2b). First, the peak at 1.4 Å is indicative of carbon content (C–C bond length in graphite = 1.42 Å). The position of this peak does not change significantly among the three MOFdCs, although the intensity differs, suggesting differences in carbon content. The first Zr–O peak appears at 2.1 Å (shortest Zr–O bond length in tetragonal and monoclinic zirconia = 2.09–2.10 Å). A shoulder on this peak at approximately 2.4 Å is observed in all three PDFs, but is more intense for UiO-67-bpy-dC. This may indicate an increased splitting related to a distortion or reduction in zirconia symmetry, as there are two Zr–O bond distances in the 2.1–2.4 Å range for tetragonal and monoclinic zirconia, but only one for cubic zirconia.^59,60^ There may also be a contribution from the second-nearest C–C distance (2.46 Å) in this peak, so the significance of the differences is unclear. The most notable difference is in the first Zr–Zr peak that appears between 3.4 and 3.6 Å. There is a notable change in both the peak position and the breadth of the peak, with MIL-140C-bpy-dC having a longer Zr–Zr distance than UiO-67-bpy-dC or Zr-ABTC-dC. This may indicate that the MIL-140C-bpy-dC has a greater contribution from high symmetry zirconia species compared to UiO-67-bpy-dC or Zr-ABTC-dC, as the first Zr–Zr distance is greater in cubic and tetragonal zirconia than in monoclinic zirconia (3.63 Å for cubic and tetragonal zirconia vs 3.35 and 3.45 Å for monoclinic zirconia).^61,62^ Finally, comparison of the experimental PDF to literature zirconia structures indicates that the zirconia species in all three MOFdCs is unlikely to be cubic, as the cubic zirconia PDF contains several peaks at high-*r* that are not present in the experimental PDF patterns (Figure 2c).

To further investigate the identity of the zirconia phases, we then performed single phase refinements of the PDF patterns for MIL-140C-bpy-dC and UiO-67-bpy-dC (Figure 3). Surprisingly, over a short distance range, the best performing fit for MIL-140C-bpy-dC is a monoclinic phase, provided that zirconium positions are allowed to move by symmetry during the refinement. Of the four phases refined (monoclinic, orthorhombic, tetragonal, and cubic), only the monoclinic phase was able to give a reasonable reproduction of the low-*r* signal, particularly regarding the Zr–Zr peak around 3.6 Å. However, over a longer distance range, the tetragonal and cubic refinements perform substantially better, even with the zirconium positions refined for the monoclinic phase. This was surprising, as it suggests that the local atomic structure differs from the average structure observed over longer distance ranges.

**Figure 3:**
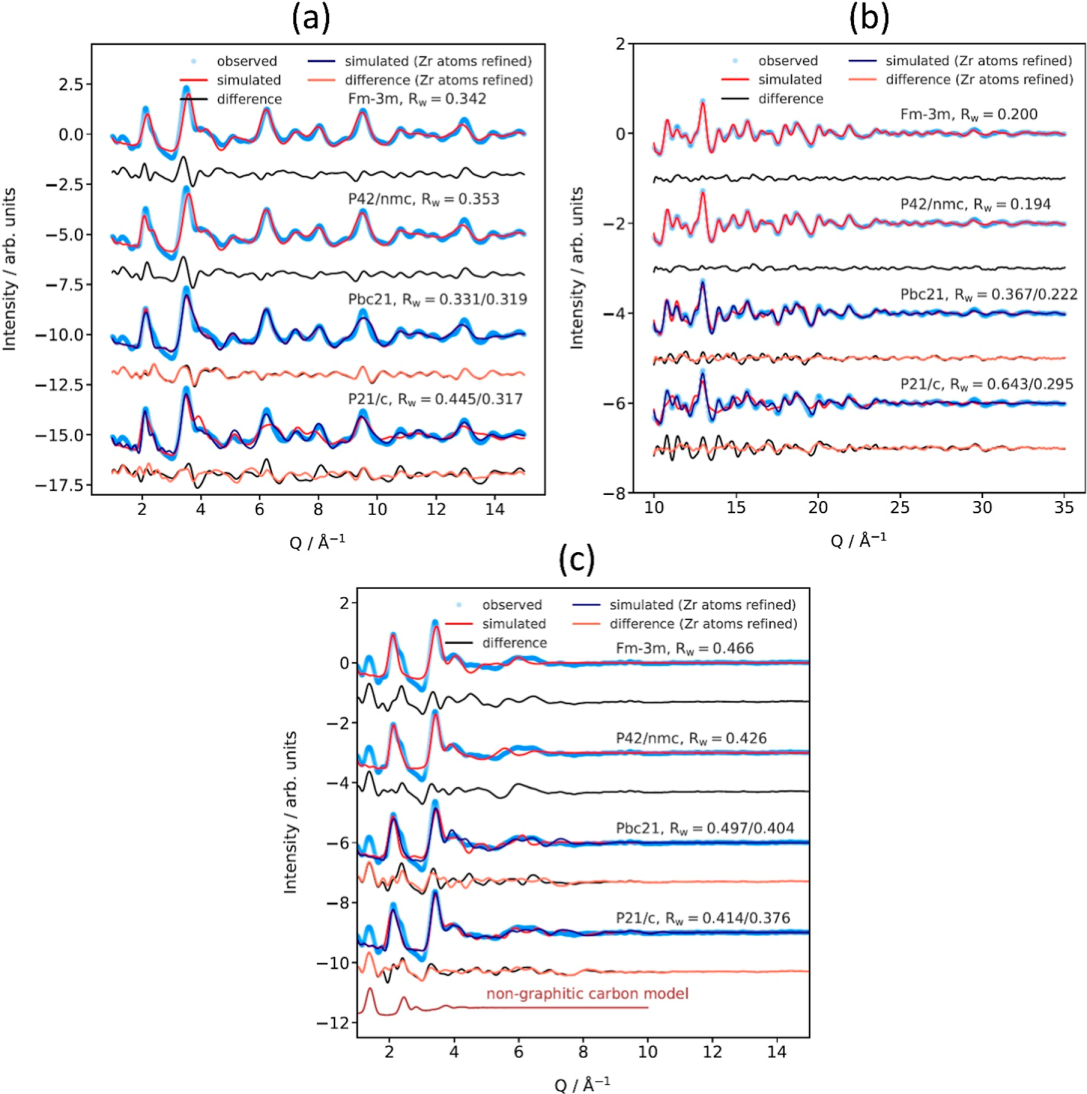
Single phase refinements of the PDF pattern of MIL-140C-bpy-dC over (a) short (1–15 Å) and (b) long (10–35 Å) distance ranges and (c) single phase refinements of the PDF pattern of UiO-67-bpy-dC over a short (1–15 Å) distance range.

For UiO-67-bpy-dC, only short distance range refinements could be conducted due to the increased damping of the signal due to smaller domain sizes. For this MOFdC, the structure refinements were significantly worse due to the smaller and more disordered nanoparticles. As with MIL-140C-bpy-dC, the monoclinic zirconia phase was the best fit for the data, which is especially apparent for the weak correlations above 4 Å. In addition to the zirconia modeling, a simulation of the first few coordination shells from a nongraphitic carbon model was compared to the data, which shows that the additional low-*r* peaks not accounted for by the zirconia phases are likely due to the carbon content, which agrees with the qualitative observations above. Although these models do not explicitly account for nitrogen doping in the carbon, the nitrogen content is sufficiently low that it is unlikely to cause a significant distortion or alteration to the carbon structure. It is also possible that the feature at 2.5 Å contains contributions from both Zr–O correlations and second-nearest C–C correlations. The differences in single phase model performance between the short- and long-range data, as well as the need to refine zirconium positions to account for structural distortions, are consistent with the qualitative observations described above.

Given the disagreement between short- and long-distance range models for single phase fits, we then modeled each MOFdC as a multiphase system, with contributions from each of monoclinic zirconia, tetragonal zirconia, and nongraphitic carbon (Figure 4, Table 2). These fits indicate that the structure of the zirconia becomes more similar to the tetragonal phase in the order UiO-67-bpy-dC < Zr-ABTC-dC < MIL-140C-bpy-dC. The fits also suggest that the ordering or size increase of the zirconia particles is associated with an increase in the apparent symmetry of the atomic structure. There is no clear trend in the mass fraction of monoclinic zirconia and both zirconia phases are of sizes 2–4 orders of magnitude smaller than the template MOF crystals, with no clear correlation between zirconia particle size and MOF crystal size. However, there were significant anticorrelations between the tetragonal and monoclinic phase scale factors for some refinements of the UiO-67-bpy-dC and Zr-ABTC-dC data, likely due to the small coherence. The ultrasmall size of the nanoparticles and the more limited order in these two MOFdCs means that the two phases are likely competing over shared features of the local coordination, limiting the reliability of the quantitative phase determination in the PDF models. An alternative 2-phase fit, where only monoclinic zirconia and graphite were considered, produces similar total zirconia content, albeit with a significantly worse fit in the case of MIL-140C-bpy-dC, which further suggests that the distinction between monoclinic and tetragonal zirconia is not well-defined. These results indicate that, while the symmetry of the IBU is not transferred to the zirconia, the prestructuring of the IBU over greater distances in MIL-140C-bpy templates the formation of higher coherence zirconia compared to the zirconia formed from the discrete Zr_6_-oxo clusters of the other two MOFs. The carbon content also varies between the MOFdCs, which agrees with the differences in observed intensity of the first C–C peak in the PDF.

**Figure 4:**
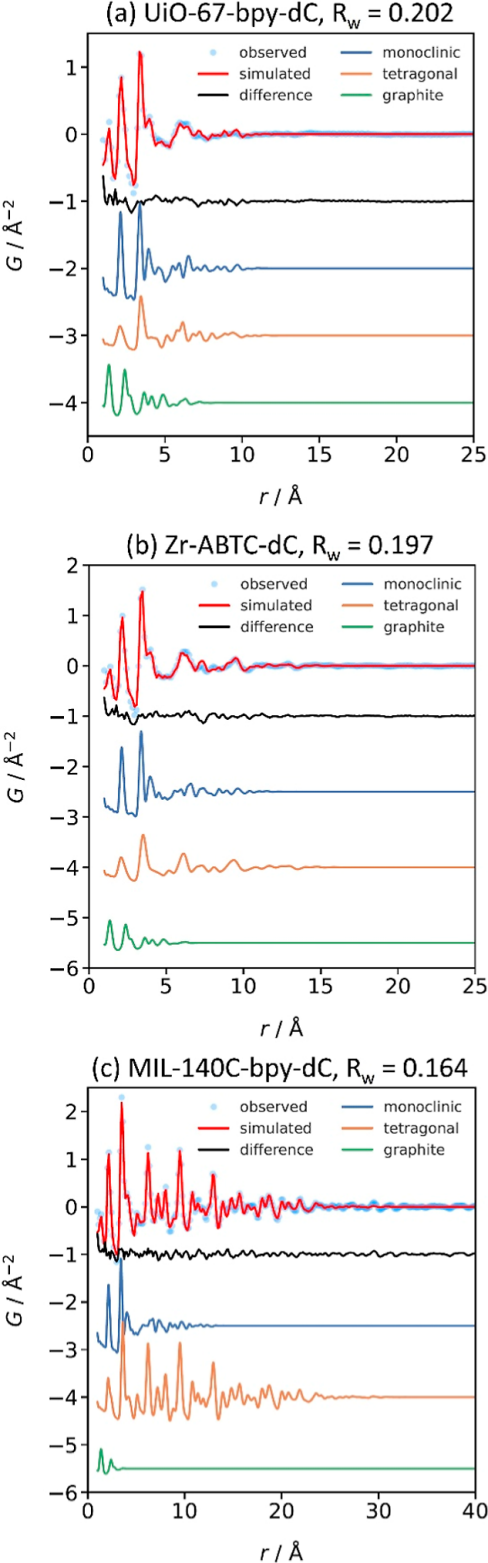
Multiphase refinements of PDF patterns for (a) UiO-67-bpy-dC, (b) Zr-ABTC-dC, and (c) MIL-140C-bpy-dC.

**Table 2:** Results of Refinements on Synchrotron PDF Data for Each MOFdC

			phase mass fraction			spherical domain size (Å)		
MOFdC	parent MOF IBU	*R*_w_	t-ZrO_2_	m-ZrO_2_	graphite	t-ZrO_2_	m-ZrO_2_	graphite
UiO-67-bpy-dC	Zr_6_-oxo clusters	0.202	0.12	0.34	0.54	13	12	9
Zr-ABTC-dC	Zr_6_-oxo clusters	0.197	0.16	0.32	0.48	17	13	8
MIL-140C-bpy-dC	extended 1D Zr-oxo chains	0.164	0.18	0.36	0.50	34	15	4

Additionally, the multiphase fits also show that the domain size of tetragonal zirconia increases dramatically as the phase mass fraction increases. This may indicate the presence of a pseudosymmetry whereby over longer distances, a local structure that appears monoclinic averages out to resemble the higher symmetry phase. As part of developing the multiphase fits, we also considered the residual of the MIL-140C-bpy-dC data after the monoclinic and graphitic components were removed (Figure S14). The monoclinic and graphitic components of the MIL-140C-bpy-dC residual bear a strong resemblance to the structure signal of UiO-67-bpy-dC, while the remaining structure signal of MIL-140C-bpy-dC is well-described by the tetragonal phase. This is indicative of a two-phase distribution, where both the tetragonal and monoclinic phases exist independently of one another, and could be explained by two competing scenarios. First, there may be some UiO-67-bpy phase impurity in the MIL-140C-bpy prior to the calcination. Upon calcination, this phase impurity forms poorly ordered zirconia would be formed from pure UiO-67-bpy. Alternatively, both phases could be produced from the MIL-140C structure. It is also possible that the presence of higher coherence zirconia signal in MIL-140C-bpy-dC is a product of incomplete calcination due to the higher decomposition temperature of the MOF. However, the absence of residual IBU signal (Figure S13) and the rapid kinetics of MOF thermal decomposition suggest that this is unlikely.^63^ Additional modeling suggests that these similarities are not solely due to distortion of the zirconia structure, as the two-phase model for the zirconia outperforms other models even when additional parameters are considered (Figures S15–S17).

Finally, we also directly visualized the zirconia particles using transmission electron microscopy. This technique is valuable in this study as it not only allows for direct visualization of crystalline domains, but also can produce visible lattice fringes representing atomic planes in the material, which can help with phase identification. As seen in Figure 5, nanosized crystalline domains representing the zirconia nanoparticles can be identified in the TEM of the MOFdCs, demonstrating the presence of local, but not long-range, order. Notably, the size of the nanoparticles varied in agreement with the observations from the X-ray total scattering and diffraction data. The average domain size increases from 2.9 ± 0.8 nm in UiO-67-bpy-dC to 3.1 ± 0.8 nm in Zr-ABTC-dC and 4.6 ± 1.1 nm for MIL-140C-bpy-dC (see Experimental Section for a description of how domains were identified and measured). As with the PDF, this suggests that the structures of the UiO-67-bpy-derived and Zr-ABTC-derived zirconia domains are similar in size, while the MIL-140C-bpy-derived domains are larger. Like the PDF models, these domain sizes are 2–4 orders of magnitude smaller than the MOF template crystals, further supporting the claim that MOF crystal size has little effect on the size of the zirconia. While the domain sizes observed via TEM are somewhat larger than the domain sizes obtained from the PDF models, the discrepancies can be explained by the anisotropic crystallites and high levels of disorder, as the particle sizes agree well with visual inspection of the total scattering data. Likewise, in situ SAXS/WAXS patterns on UiO-67-bpy exhibit a scattering feature that becomes distinct from the MOF’s (111) reflection (*Q* = 0.41 Å^–1^) at 450–475 °C, eventually resolving at *Q* = 0.27 Å^–1^ (*d* = 2.3 nm) after 1 h at 550 °C (Figure S18). The strong agreement between the quantitative PDF models and direct visualization through TEM further supports the validity of the developed models.

**Figure 5:**
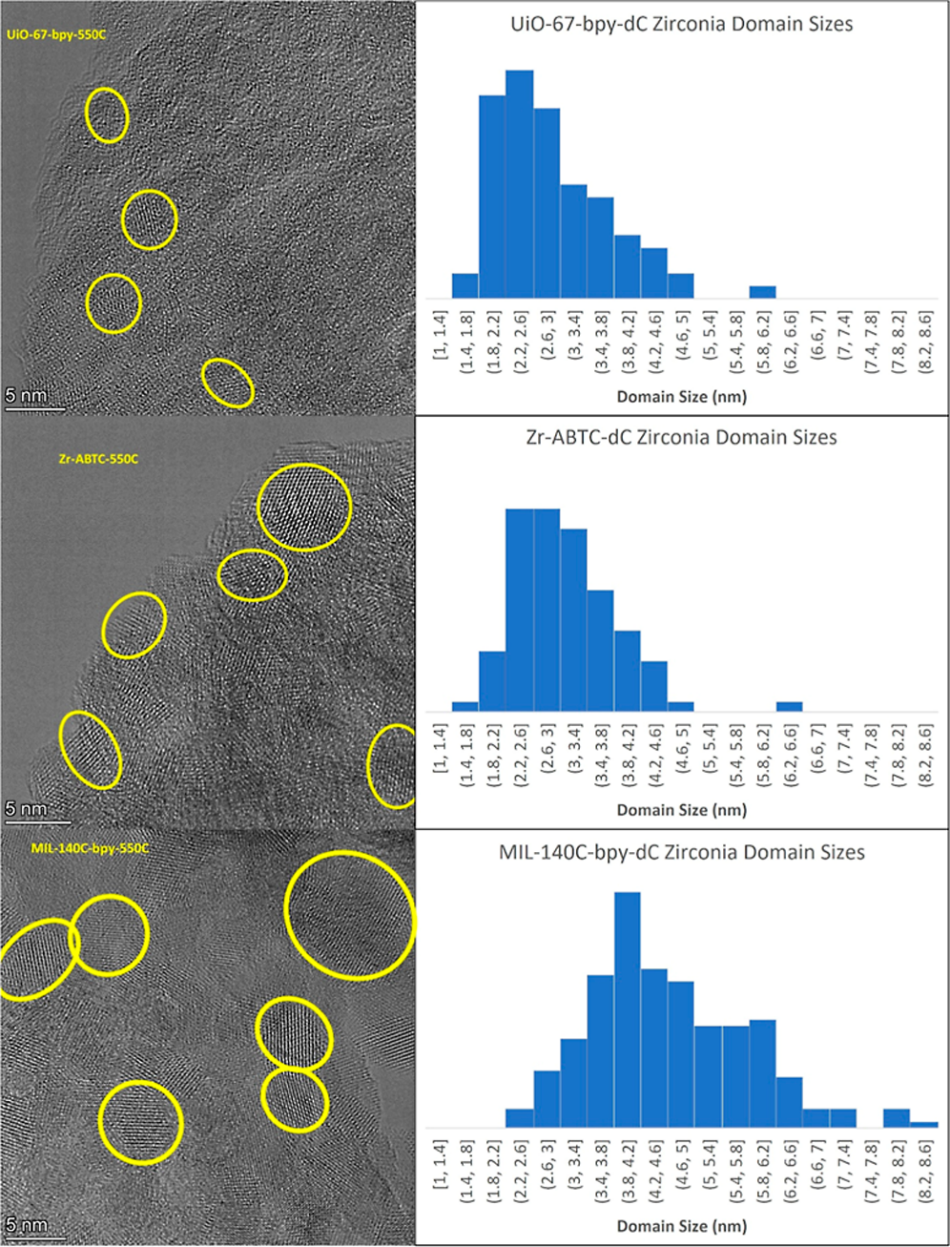
TEM images and zirconia domain size distributions for UiO-67-bpy-dC, Zr-ABTC-dC, and MIL-140C-bpy-dC. Select domains are circled in yellow.

Another question raised by this study is whether there is a limit to the MOF’s ability to template the zirconia structure. At very high temperatures (1100 °C), calcination of UiO-67-bpy produces exclusively monoclinic zirconia with large domain sizes, indicating that imparting large amounts of energy into the system will override the templation effect and favor the formation of the thermodynamic product. Likewise, progressively increasing calcination temperature of MIL-140C-bpy also produces zirconia with lower symmetry and larger domain sizes (Figures S19 and S20). Additionally, the IBU structure alone is unable to template the formation of nanoparticulate zirconia. Calcinations of zirconium oxychloride octahydrate, which crystallizes in a structure very similar to the Zr_6_-oxo clusters of UiO-67-bpy and Zr-ABTC, or zirconium tetrachloride, which exhibits an extended chain structure, both produce mixtures of monoclinic and tetragonal/cubic zirconia with much larger domain sizes (Figure S21). This suggests that some degree of overall MOF structuring is also crucial for the formation of nanoparticulate zirconia. Ongoing investigations are further studying the limits of the templation effect.

## Conclusions

Through a combination of qualitative PXRD analysis, quantitative X-ray total scattering PDF models, and direct visualization using TEM, we have analyzed the structural coherence and atomic structuring of zirconia nanoparticles derived from the carbonization of three different Zr-MOFs with different structures and IBUs. MIL-140C-bpy-derived zirconia is more structurally coherent and well-ordered at the atomic level than UiO-67-bpy- or Zr-ABTC-derived zirconia. This has been ascribed to the differing structures of the MOF IBUs, with the preorganized local atomic structure of the MIL-140C-bpy extended chain IBU exerting a templating effect on the zirconia formation. In contrast, the lack of longer-range ordering in the Zr_6_-oxo clusters of UiO-67-bpy and Zr-ABTC results in a more limited structural templation. Although the templation effect is strong at the low temperatures described in this study, ongoing work exploring the limits of the templation effect suggests that higher temperatures can override the templation by generating large domains of more thermodynamically favorable phases.

## Experimental Section

All chemicals used in this study were used as received without further purification.

### MOF Synthesis

#### UiO-67-bpy

Zirconium tetrachloride that was stored in a desiccator (670 mg) and hydrochloric acid (5 mL) were dissolved in dimethylformamide (DMF, 50 mL) ultrasonically in a 250 mL Schott bottle. Separately, 2,2′-bipyridine-5,5′-dicarboxylic acid (H_2_bpydc, 900 mg) was dissolved in DMF (100 mL) ultrasonically then added to the Schott bottle. The reaction mixture was placed in a preheated oven at 80 °C overnight. The white polycrystalline powder was washed with DMF and methanol, then dried at 80 °C overnight.

#### Zr-ABTC

Zirconium oxychloride octahydrate (32 mg) was ultrasonically dissolved in DMF (8 mL) and formic acid (6 mL) in a 20 mL glass vial, then 3,3′,5,5′-azobenzene-tetracarboxylic acid (H_4_ABTC, 36 mg) was added and ultrasonically dissolved. The reaction vial was sealed and placed in a preheated oven at 120 °C for 3 days. The orange polycrystalline powder was washed with DMF and methanol, then dried at 80 °C overnight.

#### MIL-140C-bpy

Zirconium tetrachloride that was stored in a desiccator (117 mg) and H_2_bpydc (242 mg) were ultrasonically dissolved in DMF (2.5 mL) and acetic acid (143 μL) in a 20 mL glass vial. The solution was transferred to a 10 mL autoclave, which was sealed and placed in a preheated oven at 220 °C for 12 h. It was important to remove the reaction from heat after no more than 12 h to prevent the formation of a UiO-67-bpy phase impurity. Once cooled to room temperature, the white polycrystalline powder was washed with DMF and methanol, then dried at 80 °C overnight.

***Caution!*** *The synthesis of MIL-140C-bpy is conducted at temperatures above the boiling point of the solvent. An autoclave or similar pressure vessel must be used as a reaction vessel. Ensure that the reaction vessel is cooled to room temperature before opening.*

### MOFdC Synthesis

MOFdC samples were prepared by placing approximately 30 mg of MOF in a platinum crucible. The MOFs were then calcined at 550 °C for 1 h under nitrogen in a Mettler Toledo TGA/DSC 1 equipped with a GC 200 gas controller system with a heating ramp rate of 5 K/min. After calcination, the samples were stored at standard conditions for several weeks. High temperature calcinations (>550 °C) were conducted as above or in a vertical tube furnace under argon.

### Lab-Scale X-ray Diffraction

PXRD data were collected using a Bruker D8-Focus. The X-ray source was a 2.2 kW Cu X-ray tube, maintained at an operating current of 40 kV and 25 mA. The X-ray optics was the standard Bragg–Brentano para-focusing mode with the X-ray diverging from a DS slit (1 mm) at the tube to strike the sample and then converging at a position sensitive X-ray detector (Lynx-Eye, Bruker-AXS). The two-circle 250 mm diameter goniometer was computer controlled with independent stepper motors and optical encoders for the θ and 2θ circles with the smallest angular step size of 0.0001° 2θ. The software suit for data collection and evaluation is windows based. Data collection is automated COMMANDER program by employing a DQL file. For MOF samples, the samples were ground and placed on a quartz plate or a zero-background silicon plate. Data were collected from 2° to 70° 2θ with a 0.01° step and 0.2 s step time. The PSD opening was set to 3°. For MOFdC samples, the samples were ground and placed on a zero-background silicon plate. Data were collected from 10° to 70° 2θ with a 0.06° step and 3 s step time or from 25° to 60° 2θ with a 0.06° step and 6 s step time. The PSD opening was set to 3°. Due to the large step size, additional preliminary scans were performed using the MOF data collection parameters to ensure that no narrow peaks were overlooked in the analysis.

### Synchrotron Diffraction and Total Scattering

X-ray total scattering pair distribution function (PDF) experiments were carried out at the 28-ID-1 beamline at the National Synchrotron Light Source II (NSLS-II) at Brookhaven National Laboratory using the rapid acquisition PDF method (RAPDF).^64^ A 2D PerkinElmer amorphous silicon detector was placed 200 mm behind the samples, which were ground and loaded in 1 mm outer diameter (OD) Kapton capillaries. The incident wavelength of the X-rays was 0.1867 Å and the total detector exposure time was 300 s. Calibration of the experimental setup was performed using nickel as a calibrant. Raw data were summed and corrected for polarization effects before being integrated along arcs of constant angle to produce 1D powder diffraction patterns using the program pyFAI.^65^ Correction was then made to the data and normalization was carried out to obtain the total scattering structure function, *F*(*Q*), which was Fourier transformed to obtain the pair distribution function (PDF) using PDFgetX3 ^66^ within xPDFsuite.^67^ The range of momentum transfer used in the Fourier transformation was *Q*_min_–*Q*_max_ = 0.5–24.0 Å. The modeling was carried out using GSAS-II^68^ and PDFgui.^69^

### Rietveld Modeling

Rietveld refinement of the X-ray data was attempted using the GSAS-II program.^68^ For refinements of lab scale data, the default instrument parameters for lab scale Cu K_α_ radiation were used. For synchrotron data, the instrument parameter file for the beamline (NSLS-II 28-ID-1) was used. Background was modeled as a 3-coefficient Chebyshev polynomial. Patterns were refined with an analytic Hessian type refinement with the sum of the phase fractions constrained to equal 1. The unit cells were refined and the refined unit cells were used in the final model where the cell parameters did not deviate more than 10% from the published structures. Domain sizes were typically fixed at 5 nm for initial refinements, then refined isotropically. All atoms were treated as isotropic due to the poor data quality. Due to the poor crystallinity and potential for overlapping peaks, refinement strategies were based the guidelines outlined by Rowles.^53^ Patterns for MOFdCs were initially refined against all three zirconia phases (cubic, tetragonal, and monoclinic). The patterns were later rerefined against only the tetragonal and monoclinic phases as the PDF data indicated an absence of cubic-type structuring. Alternative single-phase models were also considered, however these models exhibited poorer fits than the two-phase models. For MIL-140C-dC, an unknown contaminant resulted in several unassigned peaks. These peaks could not be identified but resemble a simple high symmetry structure. The intensity of these peaks is sufficiently small as to not significantly affect the final models.

### Pair Distribution Function Modeling

Pair distribution function analysis was performed using the program PDFgui.^69^ The final multiphase structural refinements were performed over a range of 1.0–50.0 Å on a Nyquist grid with tetragonal (*a* = *b*, *c*, scale, δ_1_, spdiameter, *U*_iso_(Zr), *U*_iso_(O)) and monoclinic (*a*, *b*, *c*, β, scale, δ_1_, spdiameter, *x*_Zr_, *y*_Zr_, *z*_Zr_, *U*_iso_(Zr), *U*_iso_(O)) phases to describe the ZrO_2_ particles and graphite (*a* = *b*, scale, spdiameter, *U*_11_ = *U*_22_ = 0.008 Å^2^, *U*_33_ = 1.0 Å^2^) to describe the carbon component. The values of *Q*_damp_ = 0.0336 Å^–1^ and *Q*_broad_ = 0.0120 Å^–1^ were determined by refinement of the Ni reference sample. Additional reference PDF signals were simulated using either PDFgui or Diffpy-CMI.^70^ A summary of alternative PDF models for each MOF can be found in Table S2 and Figures S15–S17.

### Transmission Electron Microscopy

Transmission electron microscopy (TEM) images were performed on a Titan Themis3 300 transition electron microscope with an electron acceleration voltage of 300 kV. Domains were identified visually by observing the lattice fringes and measured using ImageJ.^71^ The outer bounds of each domain were marked to identify the shape of each domain. As most domains were ellipsoid in shape, two measurements were taken for each domain: one along the short axis of the domain, and one along the long axis of the domain, each running through the approximate center of the domain. Markers were placed on each domain after measurement to ensure each domain was only measured once. The reported domain sizes are the average of the two measurements. A total of 81 domains were identified for UiO-67-bpy-dC across 8 images; a total of 94 domains were identified for Zr-ABTC-dC across 8 images; and a total of 203 domains were identified for MIL-140C-dC across 20 images.

## Supplementary Material



## Notes

Ligand synthesis, additional characterization data, alternative PDF models (PDF)
