# Supplementary material for: Structural Templation of MOF-Derived Zirconia Nanoparticles
Source: ACS Appl Mater Interfaces. 2025 Nov 14;17(47):64842–52. doi: 10.1021/acsami.5c14965 (PMC12673521; doi:10.1021/acsami.5c14965)
Supplement: Supplementary file 1 [file am5c14965_si_001.pdf]

Supporting Information

Structural Templation of MOF-Derived Zirconia Nanoparticles

Joshua A. Powell,[a, b] Maxwell W. Terban,[c] Jiaqi Zhang,[a] Songsheng Tao,[d] Jingwei Hou,[b] Simon J. L. Billinge, [d] and Hong-Cai Zhou* [a, e]

a) *Department of Chemistry, Texas A&M University, College Station TX 77843, United States.*

b) *School of Chemical Engineering, The University of Queensland, St Lucia, 4072, Australia.*

c) *Max Planck Institute for Solid State Research, Heisenbergstr. 1, 70569 Stuttgart, Germany*

d) *Department of Applied Physics and Applied Mathematics, Columbia University, New York, New York 10027, United States*

e) *Department of Materials Science and Engineering, Texas A&M University, College Station TX 77843, United States.*

** Email: zhou@chem.tamu.edu*

Ligand Synthesis

H_2bpydc Potassium permanganate (39.0 g, 247 mmol) was dissolved in 250 mL of distilled water. 5,5'-Dimethyl-2,2'-bipyridine (6.99 g, 37.9 mmol) was added and the solution heated to 115 °C for 2 h. The solution was cooled to room temperature, filtered through celite, then the filtrate was cooled to 4 °C. The filtrate was acidified with 1 M hydrochloric acid and the pale blue precipitate was collected and washed with cold water. The precipitate was dried, then refluxed in acetone to remove impurities. The suspension was hot filtered and the solid was dried overnight under vacuum. Yield 5.95 g, 63%. ^{1}H NMR (400 MHz, DMSO-d_6): δ 9.19 (s, 2H), 8.56 (d, J = 8.3 Hz, 2H), 8.44 (d, J = 8.3 Hz, 2H)

H_4ABTC An aqueous solution of 5-nitroisophthalic acid (2.00 g, 9.47 mmol) and sodium hydroxide (4.01 g, 100. mmol) in 25 mL of distilled water was heated to 70 °C and bubbled with air for 1 h. An aqueous solution of glucose (4.00 g, 22.2 mmol) in 25 mL water was slowly added to the first solution and the resulting solution was stirred overnight at 70 °C while bubbling with air. The solution was cooled to 60 °C and concentrated under reduced pressure. The solution was further cooled to room temperature and the yellow precipitate was collected by vacuum filtration. The precipitate was subsequently dissolved in water and acidified with concentrated hydrochloric acid to produce an orange powder, which was collected by vacuum filtration. Yield 1.22 g, 72%. ^{1}H NMR (400 MHz, DMSO-d_6): δ 13.65 (bs, 4H), 8.65 (s, 6 H).

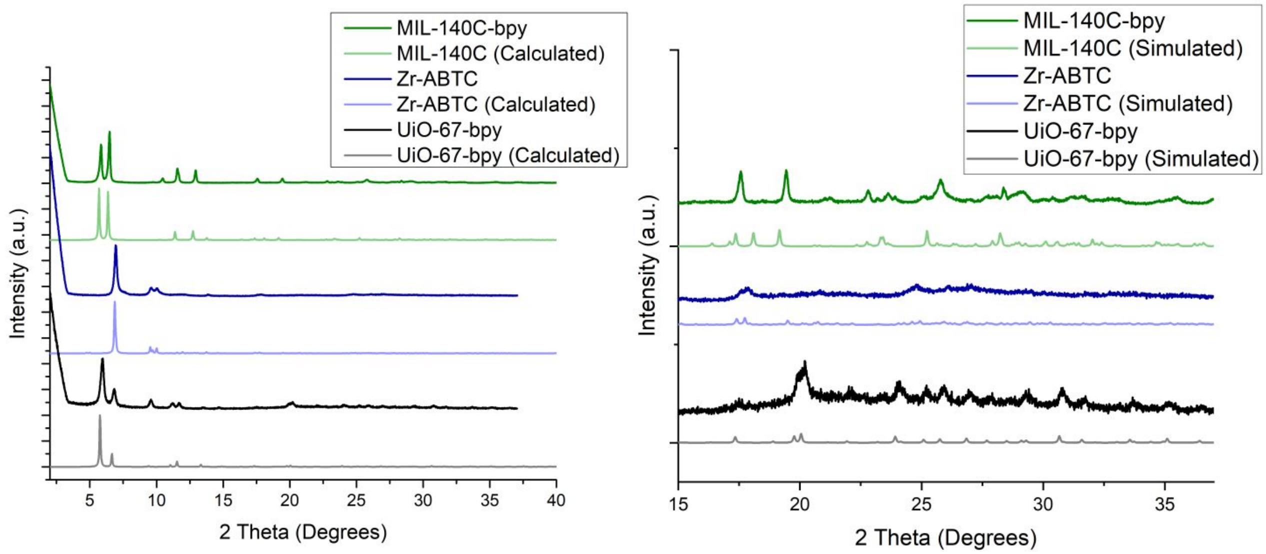

Figure S1: PXRD patterns of template MOF structures UiO-67-bpy, Zr-ABTC, and MIL-140C showing good matches with literature structures, characteristic low angle MOF peaks, and the absence of coherent zirconia (~30° 2-theta).

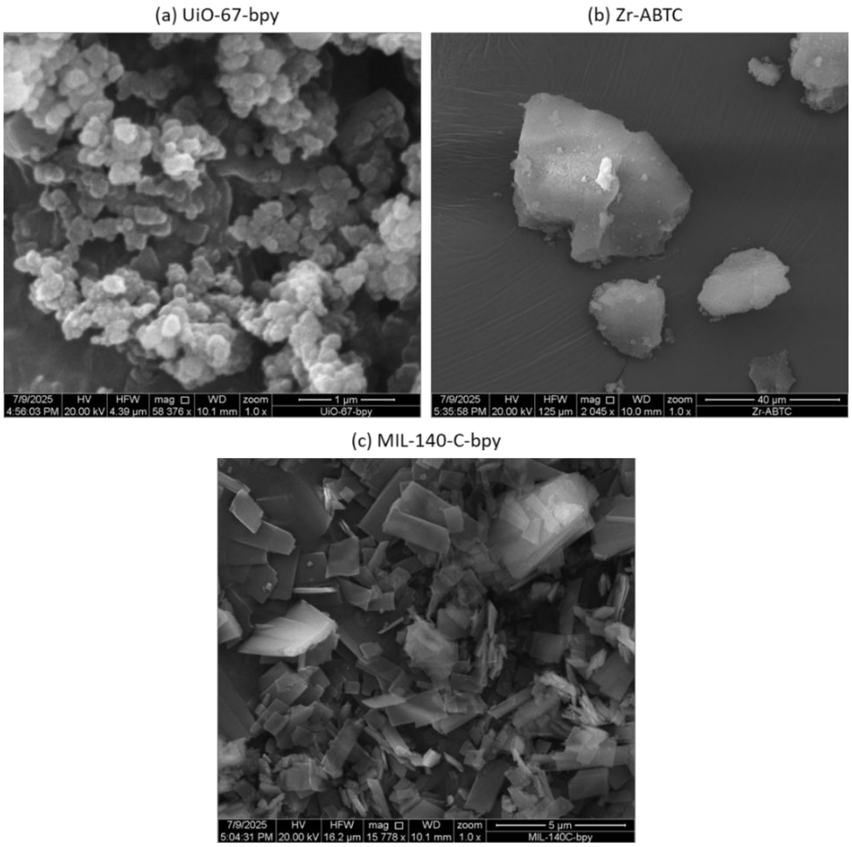

Figure S2: SEM of template MOF crystallites (a) UiO-67-bpy, (b) Zr-ABTC, and (c) MIL-140C.

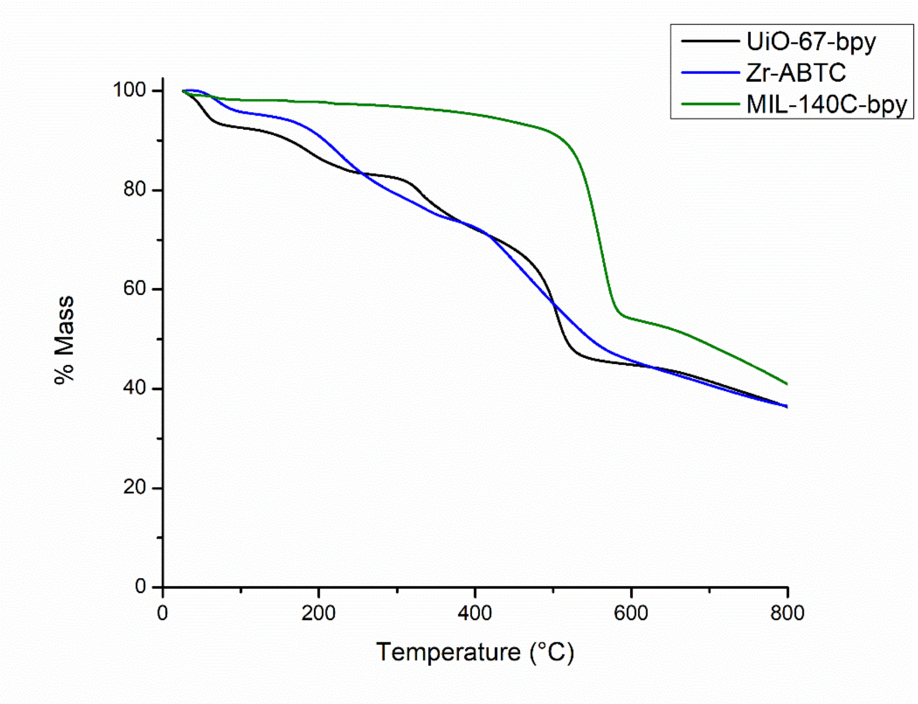

Figure S3: Thermogravimetric analysis curves for UiO-67-bpy, Zr-ABTC, and MIL-140C.

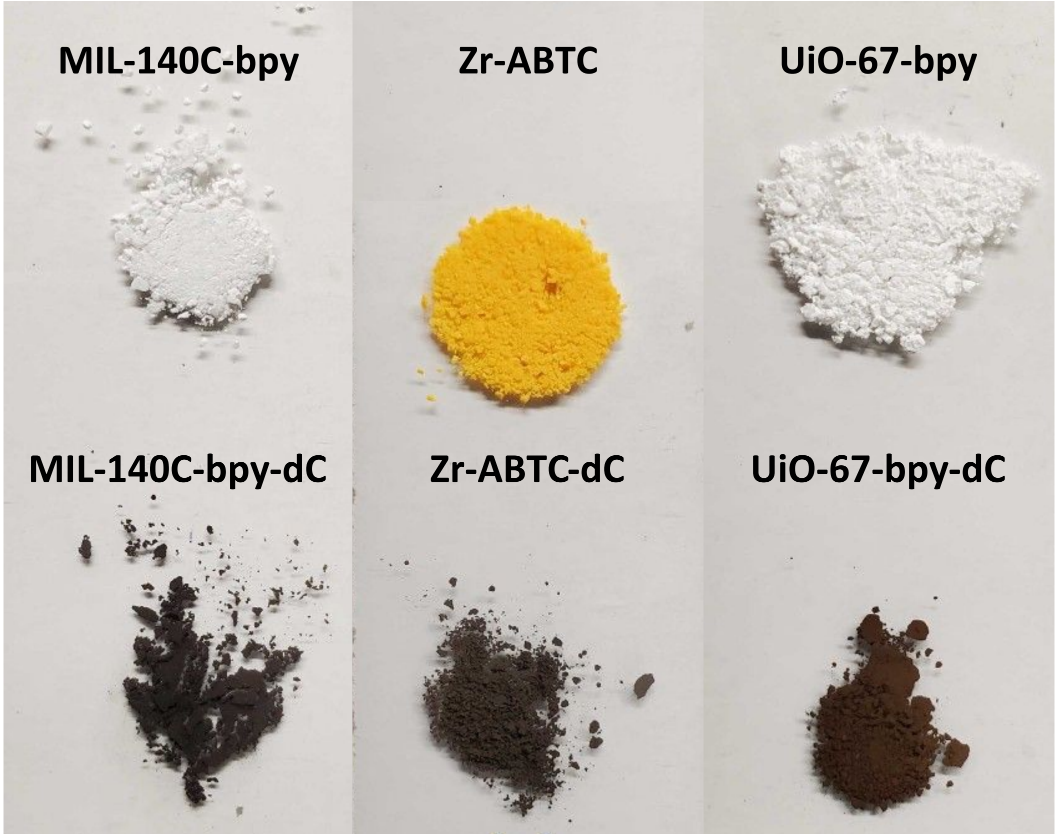

Figure S4: Photographs of template MOFs UiO-67-bpy, Zr-ABTC, and MIL-140C-bpy and their respective MOF-derived carbons.

Table S1: Elemental microanalysis of MOFdCs.

MOFdC	Carbon Content (wt%)	Nitrogen Content (wt%)	C:N Ratio (wt/wt)
UiO-67-bpy-dC	28.85	6.04	4.77 : 1
Zr-ABTC-dC	26.94	2.97	9.07 : 1
MIL-140C-bpy-dC	33.26	6.88	4.83 : 1

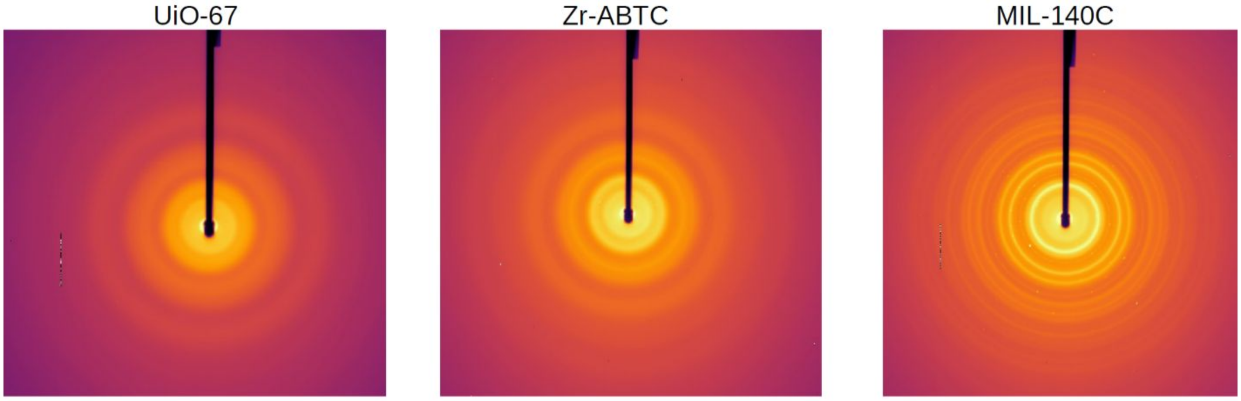

Figure S5: 2-Dimensional diffraction intensities of MOFdCs prior to integration.

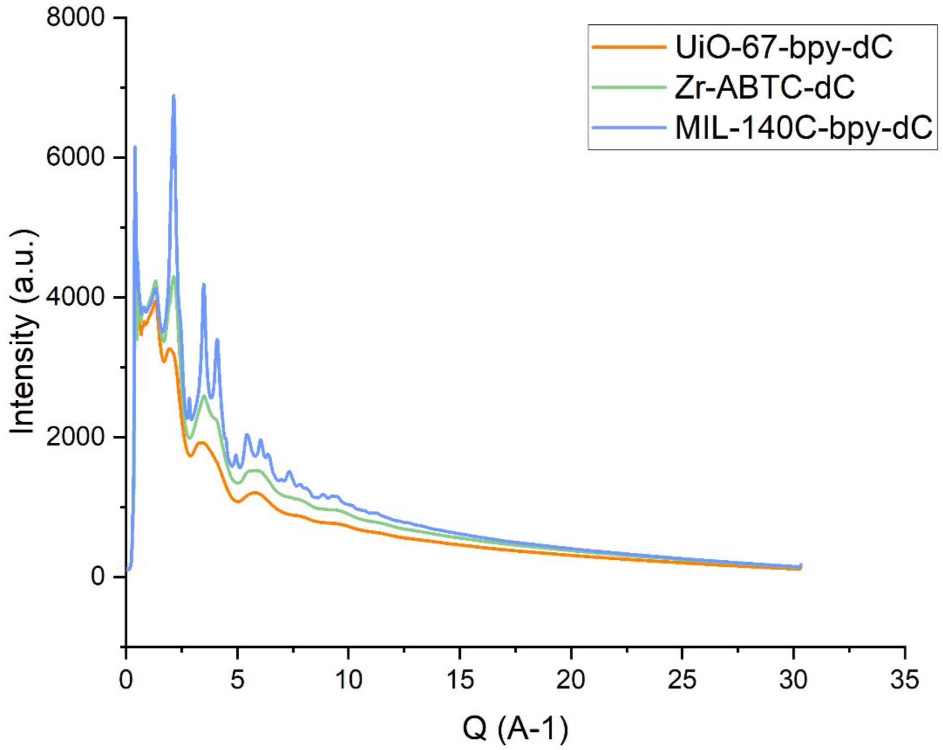

Figure S6: Integrated synchrotron X-ray diffraction intensities for MOFdCs.

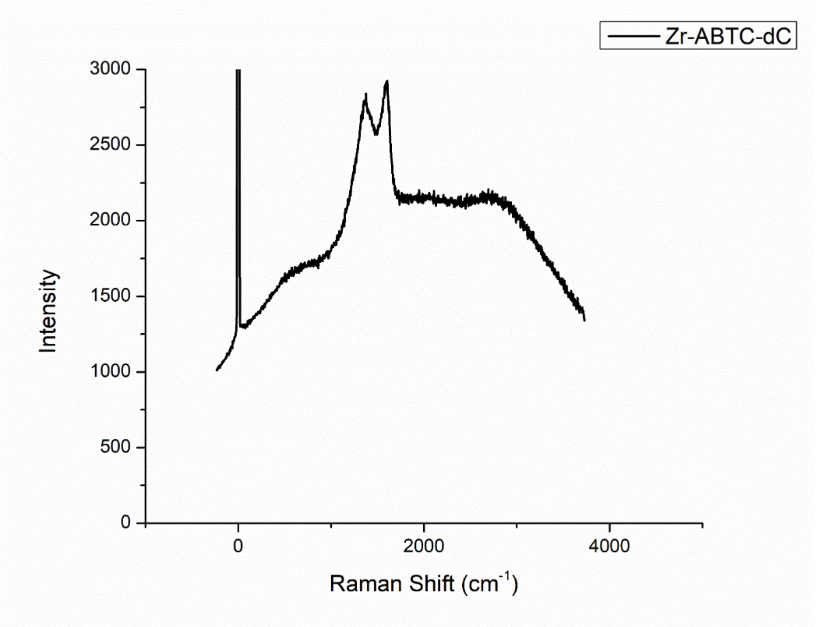

Figure S7: Raman spectrum of Zr-ABTC-dC, demonstrating the unsuitability of the technique for zirconia phase identification in MOFdCs. No zirconia absorption was observed due to the strongly absorbing carbon.

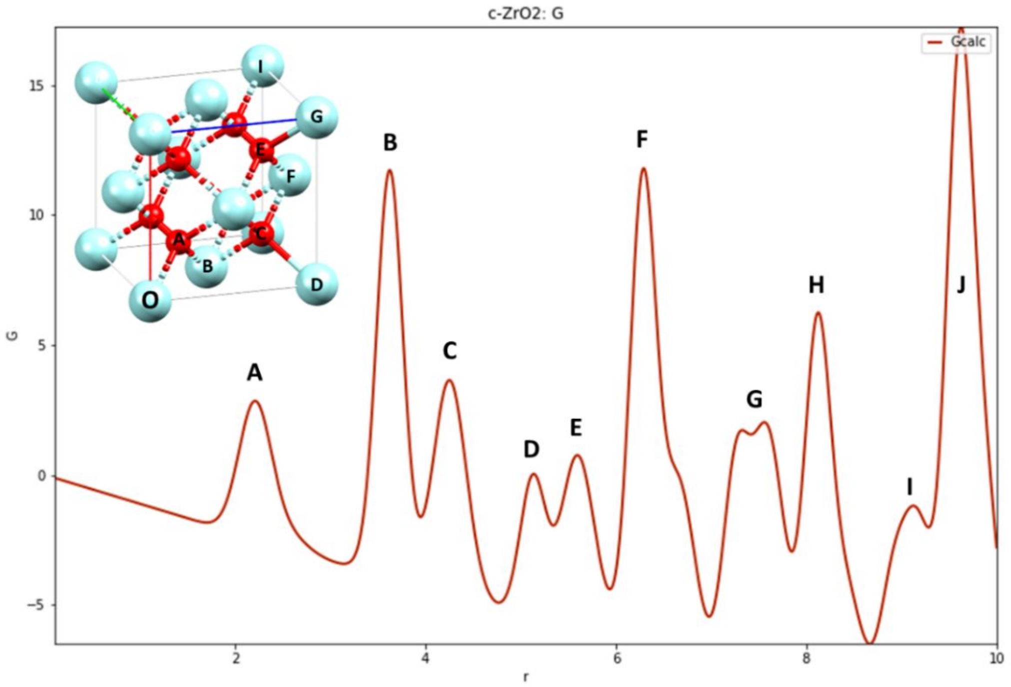

Figure S8: Calculated PDF of c-ZrO_2 with peak assignments relative to Atom “O”. Note that peaks H and J and a component of peak G represent atoms in a neighbouring unit cell, and are therefore not shown on the structure. Note that assigned peaks may have contributions from other atom-atom pairs of similar distances.

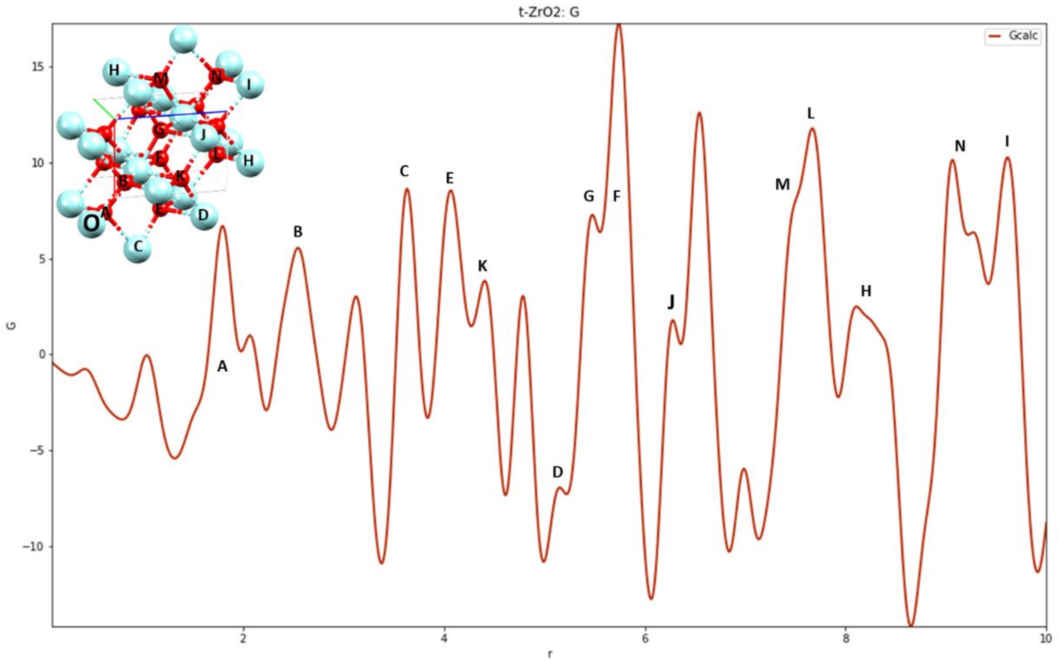

Figure S9: Calculated PDF of t-ZrO_2 with peak assignments relative to Atom “O”. Unassigned peaks are typically related to oxygen-oxygen pairs. Note that assigned peaks may have contributions from other atom-atom pairs of similar distances.

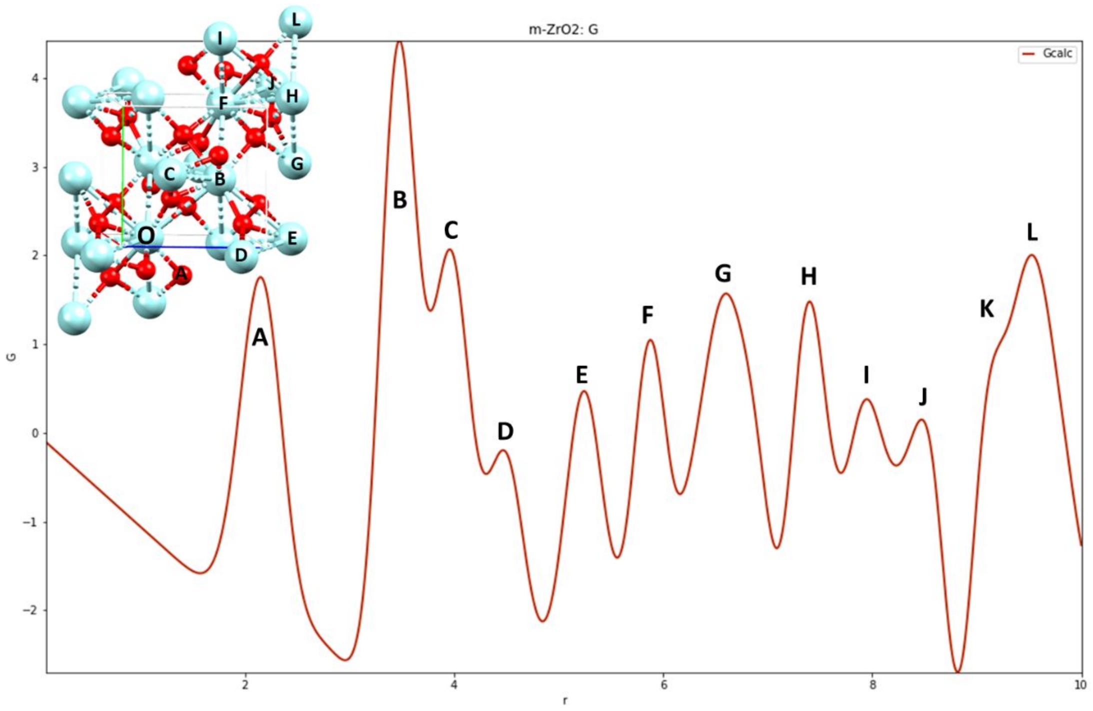

Figure S10: Calculated PDF of m-ZrO_2 with peak assignments relative to Atom "O". Note that Peak K represents an atom in a neighbouring unit cell, and is therefore not shown on the structure. Note that assigned peaks may have contributions from other atom-atom pairs of similar distances.

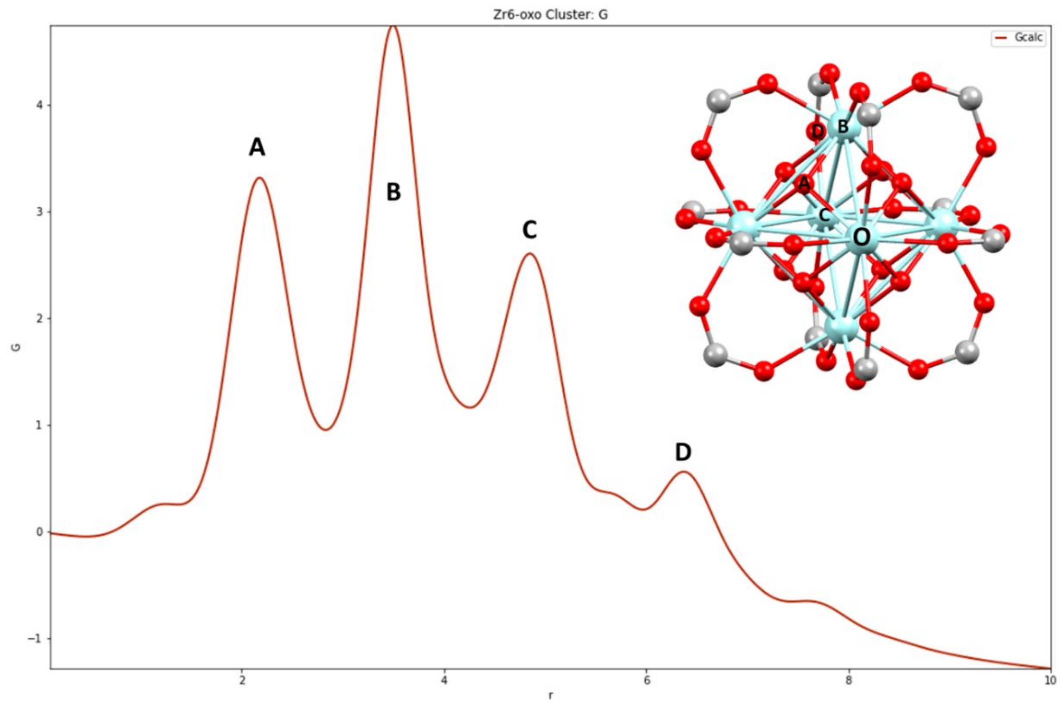

Figure S11: Calculated PDF of Zr_6-oxo cluster with peak assignments relative to Atom "O". Minor unassigned peaks correspond to oxygen-oxygen or oxygen-carbon pairs.

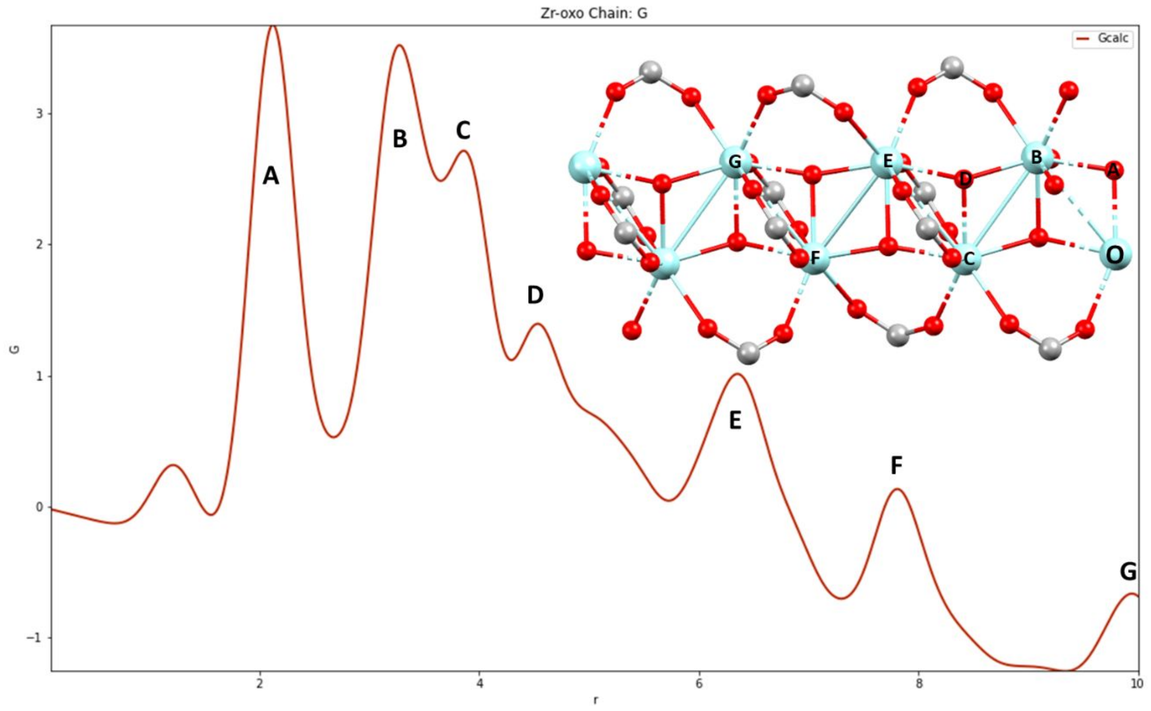

Figure S12: Calculated PDF of Zr-oxo chains with peak assignments relative to Atom “O”. Minor unassigned peaks correspond to oxygen-oxygen or oxygen-carbon pairs.

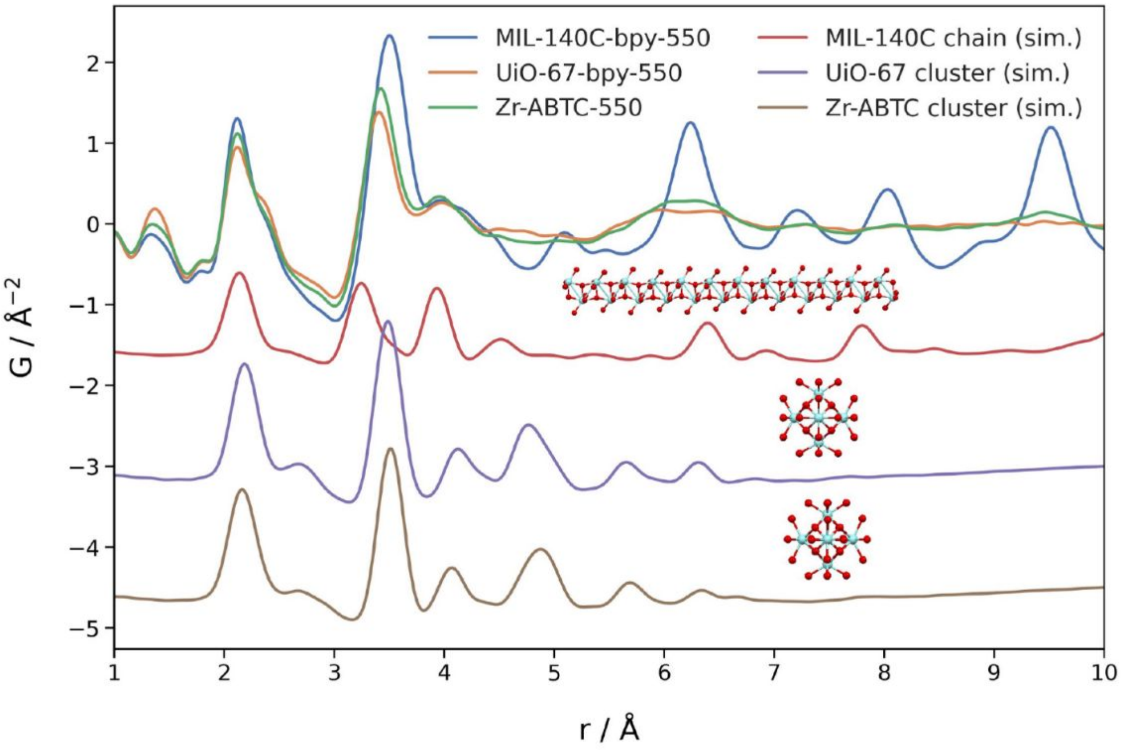

Figure S13: Comparison of pair distribution functions of MOFdCs and simulated inorganic building units for each template MOF.

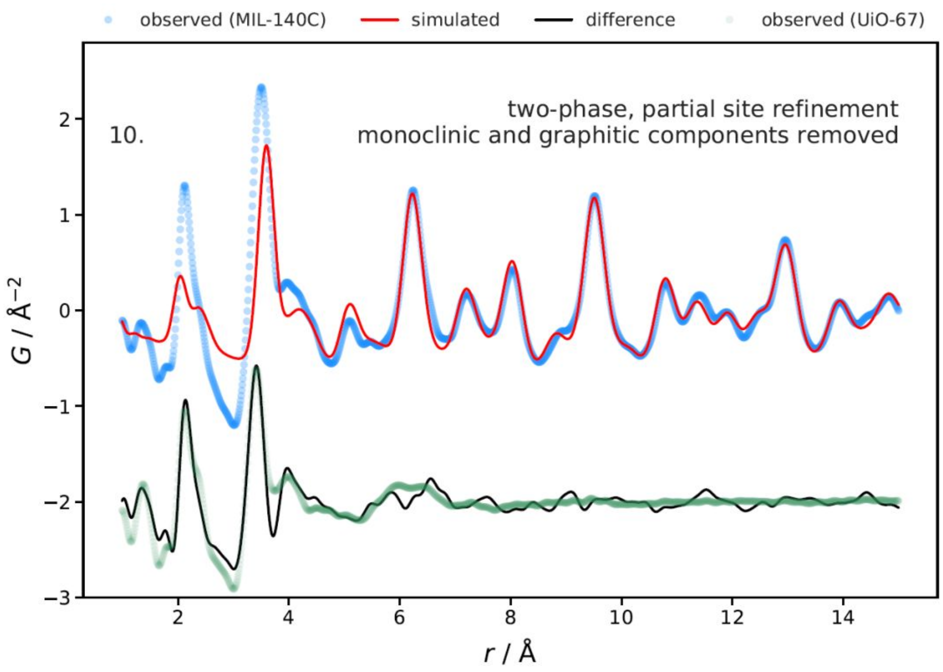

Figure S14. Residual of MIL-140C-bpy-dC PDF with monoclinic and graphitic components removed (red) compared to experimental MIL-140C-bpy-dC PDF (blue). Simulation of monoclinic and graphitic components (black) is overlaid on the experimental UiO-67-bpy-dC PDF (green).

Table S2. Alternative PDF models considered for each MOFdC.

**Model**	**Phase**	***Parameters Refined***
1. Cubic (*Fm-3m*), 9 parameters	ZrO_2	*a,* scale, *δ1*, spdiameter, U_{iso}(Zr), U_{iso}(O)
	Graphite	*a*, scale, spdiameter
2. Tetragonal ($P4_2/nmc$), 10 parameters	ZrO_2	*a, c,* scale, *δ1*, spdiameter, U_{iso}(Zr), U_{iso}(O)
	Graphite	*a*, scale, spdiameter
3. Tetragonal ($P4_2/nmc$), sites refined, 11 parameters	ZrO_2	*a, c,* scale, *δ1*, spdiameter, U_{iso}(Zr), U_{iso}(O), 1 O*(z)*
	Graphite	*a*, scale, spdiameter
4. Distorted tetragonal (*P1*), starting from $P4_2/nmc$ model, 29 parameters	ZrO_2	*a, b, c,* scale, *δ1*, spdiameter, U_{iso}(Zr), U_{iso}(O), 2 Zr(*x,y,z*), 4 O(*x,y,z*)
	Graphite	*a*, scale, spdiameter
5. Orthorhombic ($Pbc2_1$), 11 parameters	ZrO_2	*a, b, c,* scale, *δ1*, spdiameter, U_{iso}(Zr), U_{iso}(O)
	Graphite	*a*, scale, spdiameter
6. Orthorhombic ($Pbc2_1$), sites refined, 20 parameters	ZrO_2	*a, b, c,* scale, *δ1*, spdiameter, U_{iso}(Zr), U_{iso}(O), 1 Zr(*x,y,z*), 2 O(*x,y,z*)
	Graphite	*a*, scale, spdiameter
7. Monoclinic ($P2_1/c$), 12 parameters	ZrO_2	*a, b, c, β,* scale, *δ1*, spdiameter, U_{iso}(Zr), U_{iso}(O)
	Graphite	*a*, scale, spdiameter
8. Monoclinic ($P2_1/c$), sites refined, 21 parameters	ZrO_2	*a, b, c, β,* scale, *δ1*, spdiameter, U_{iso}(Zr), U_{iso}(O), 1 Zr(*x,y,z*), 2 O(*x,y,z*)
	Graphite	*a*, scale, spdiameter
9. Two-phase: Tetragonal plus Monoclinic, 18 parameters	ZrO_2 Tetrag.	*a, c,* scale, *δ1*, spdiameter, U_{iso}(Zr), U_{iso}(O)
	ZrO_2 Mono.	*a, b, c, β,* scale, *δ1*, spdiameter, U_{iso}(Zr), U_{iso}(O)
	Graphite	*a*, scale, spdiameter
10. Two-phase Tetragonal plus Monoclinic, partial site refinement, 22 parameters	ZrO_2 Tetrag.	*a, c,* scale, *δ1*, spdiameter, U_{iso}(Zr), U_{iso}(O), 1 O(*x,y,z*)
	ZrO_2 Mono.	*a, b, c, β, scale, δ1, spdiameter,* U_{iso}*(Zr),* U_{iso}*(O), 1 Zr(x,y,z)*
	Graphite	*a*, scale, spdiameter

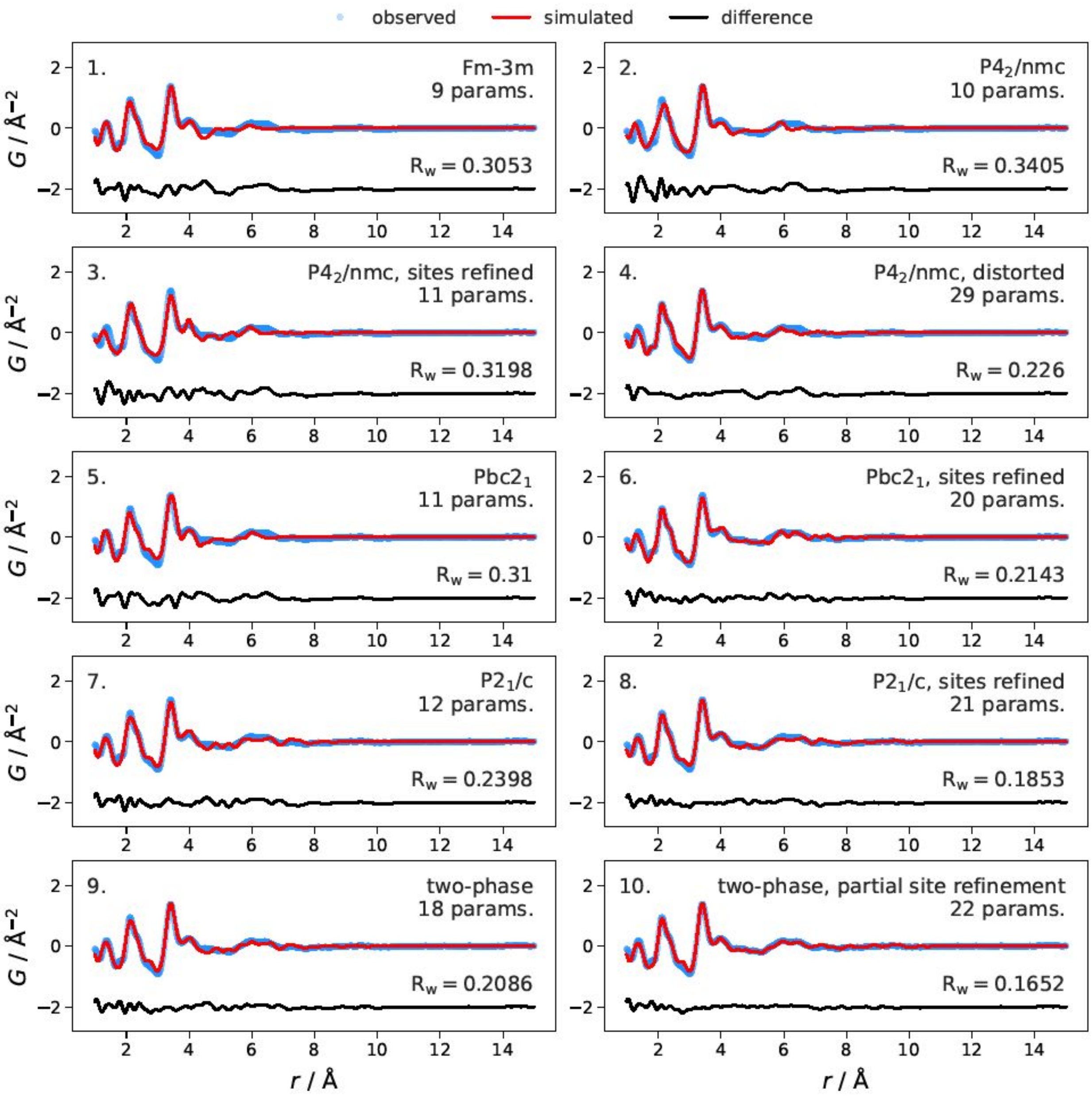

Figure S15. Alternative PDF models for UiO-67-bpy-dC.

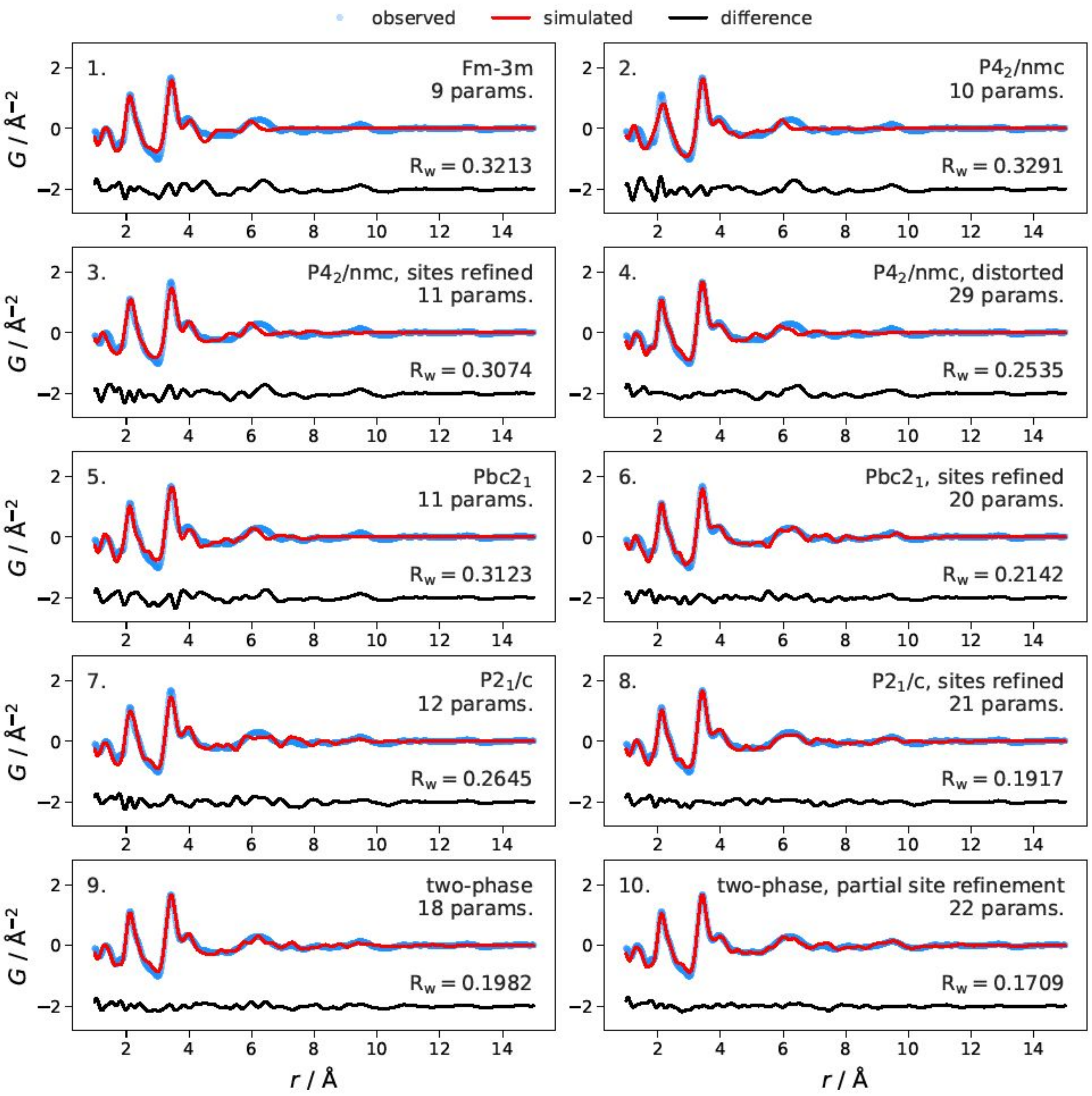

Figure S16. Alternative PDF models for Zr-ABTC-dC.

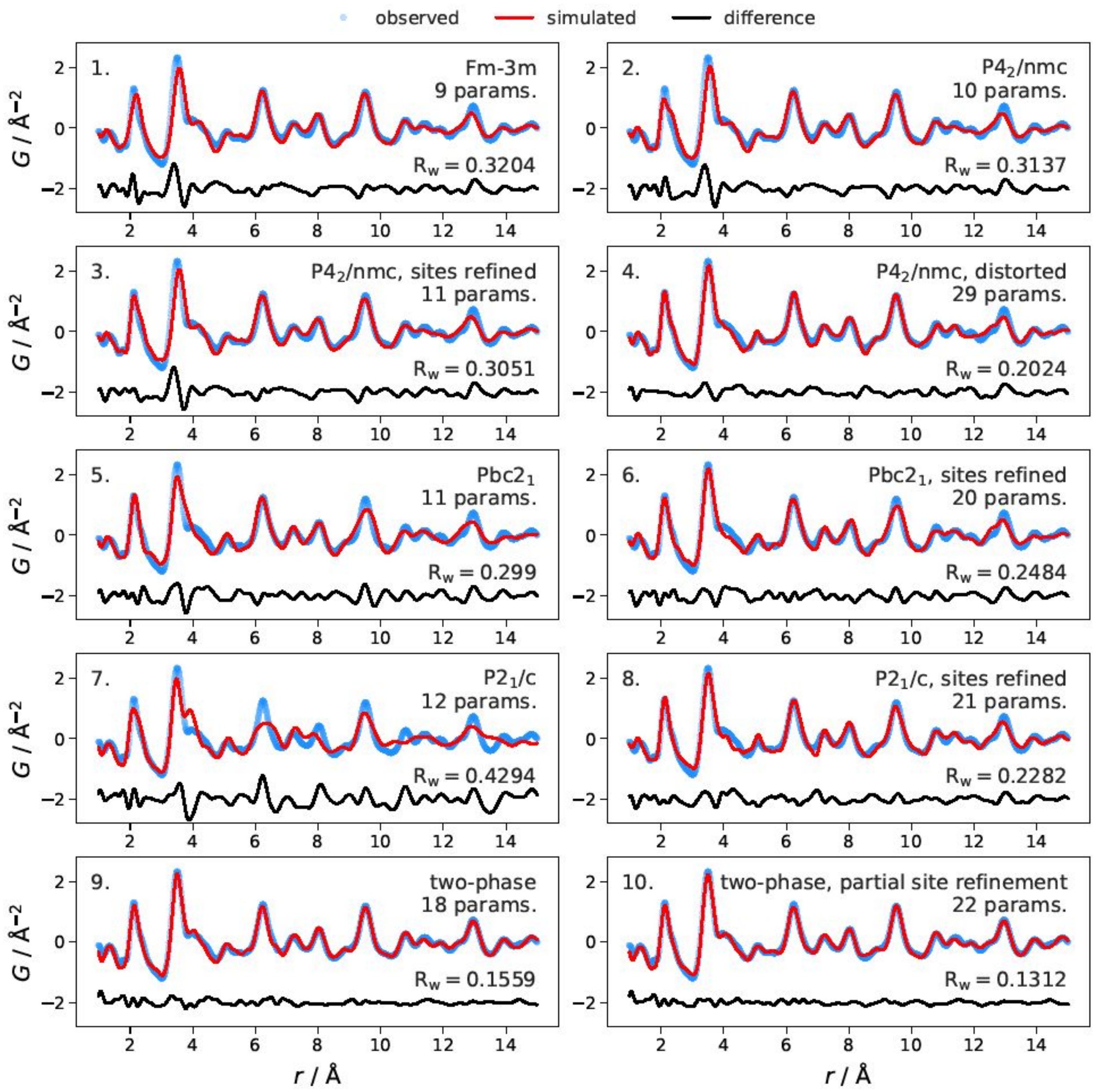

Figure S17. Alternative PDF models for MIL-140C-dC.

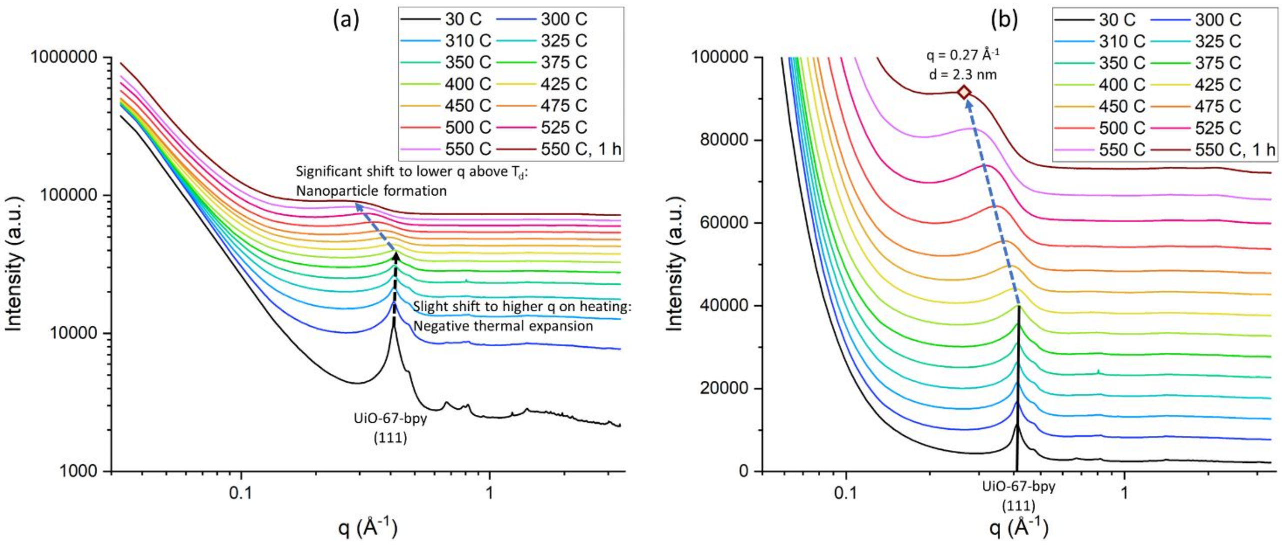

Figure S18: (a, b) In situ SAXS/WAXS of UiO-67-bpy showing negative thermal expansion of the lattice[1] below T_d and nanoparticle formation above T_d (d = 2.3 nm after 1 h at 550 °C).

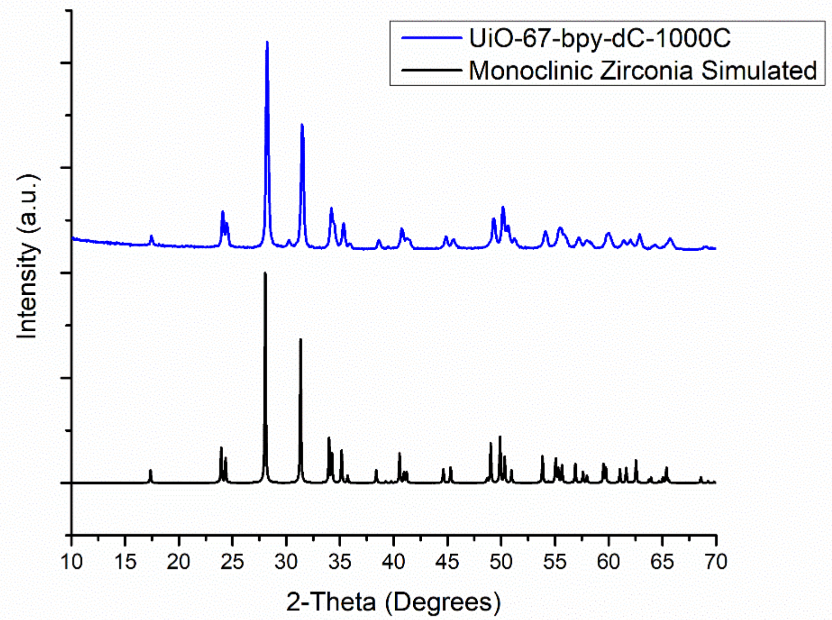

Figure S19: PXRD of UiO-67-bpy calcined at 1000 °C showing only monoclinic zirconia with large domain sizes.

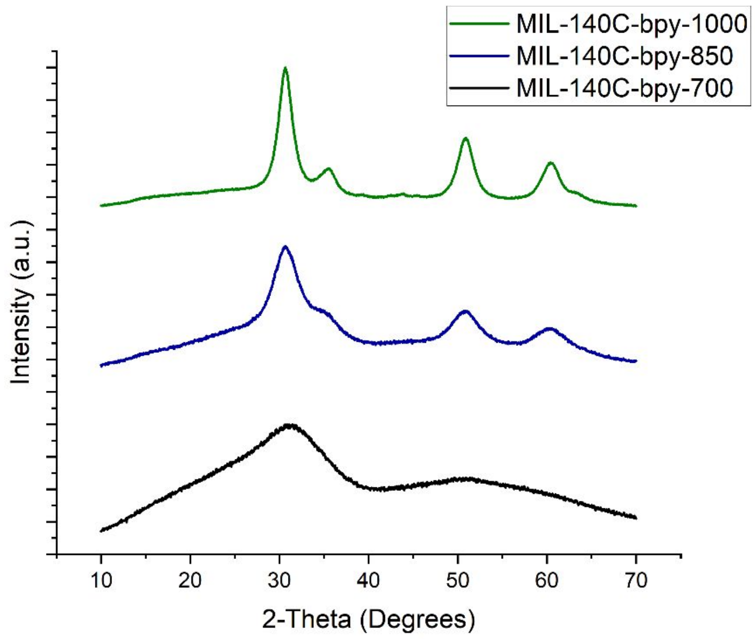

Figure S20: PXRD of MIL-140C-bpy calcined at various temperatures showing decrease in zirconia symmetry at higher temperatures.

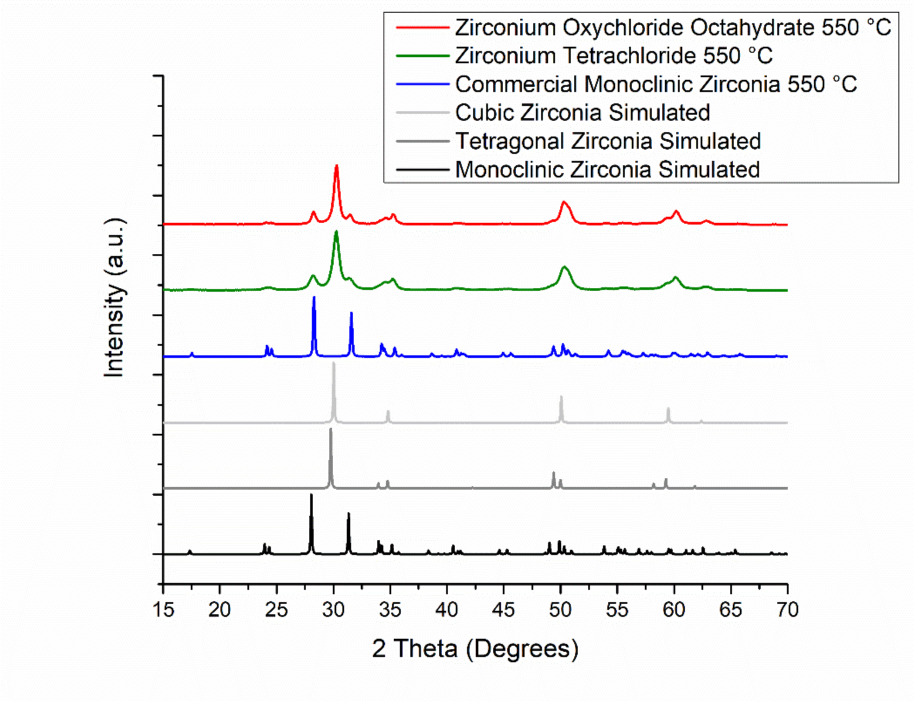

Figure S21: PXRD of calcined Zr sources.

References

(1) Goodenough, I.; Devulapalli, V. S. D.; Xu, W.; Boyanich, M. C.; Luo, T.-Y.; De Souza, M.; Richard, M.; Rosi, N. L.; Borguet, E. Interplay between Intrinsic Thermal Stability and Expansion Properties of Functionalized UiO-67 Metal–Organic Frameworks. *Chem. Mater.* **2021**, *33* (3), 910-920. DOI: 10.1021/acs.chemmater.0c03889.
